# *LILAR*, a novel long noncoding RNA regulating autophagy in the liver tissues of endotoxemic mice through a competing endogenous RNA mechanism

**DOI:** 10.1002/mco2.398

**Published:** 2023-10-10

**Authors:** Tian Tian, Shan Li, Haihua Luo, Yijing Li, Hanghang Chen, Ying Yang, Guangqin Chen, Bingyao Xie, Zhengzheng Yan, Zhenqi Wang, Lei Li, Yong Jiang

**Affiliations:** ^1^ Guangdong Provincial Key Laboratory of Proteomics State Key Laboratory of Organ Failure Research Department of Pathophysiology School of Basic Medical Sciences Southern Medical University Guangzhou Guangdong China

**Keywords:** autophagy, endotoxemia, liver injury, long noncoding RNA, microRNA

## Abstract

Sepsis is an often‐deadly complication of infection that can lead to multiple organ failure. Previous studies have demonstrated that autophagy has a protective effect on liver injury in sepsis. Here, we report a novel long noncoding RNA (lncRNA), named lipopolysaccharide (LPS)‐induced liver autophagy regulator (*LILAR*), which was highly induced in the liver tissues of endotoxemic mice. *LILAR* deficiency significantly increased the susceptibility of mice to LPS. In contrast, *LILAR* overexpression rescued the liver injury mediated by *LILAR* deficiency and increased the survival of *LILAR* knockout mice with endotoxemia. Autophagy‐related protein 13 (Atg13) is a potential downstream target gene of *LILAR*. *LILAR* deficiency notably decreased Atg13 expression and suppressed autophagy in the livers of mice challenged with LPS. A reporter gene assay showed that *LILAR* competitively adsorbed miR‐705 to increase the expression of Atg13 in cultured cells, indicating that *LILAR* participates in the regulation of the autophagy in the liver tissues of endotoxemic mice through a competitive endogenous RNA mechanism. In summary, we identified a novel lncRNA, *LILAR*, as a hepatic autophagy regulator, which not only promotes our understanding of liver pathophysiology but also provides a potential therapeutic target and/or diagnostic biomarker for liver injury in endotoxemia.

## Graphical abstract

*LILAR*, an lncRNA induced by LPS in the liver, acts as a sponge to release the suppressive miR‐705 bound to the 3′‐untranslated region of Atg13, thereby enhancing Atg13 expression. As a consequence, *LILAR* promotes autophagy activity in the liver, ultimately reducing liver injury in mice. Conversely, *LILAR* deficiency downregulates Atg13 and hampers autophagy activity, resulting in increased mouse mortality rates following LPS injection.

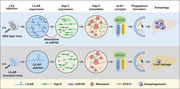

## 1 INTRODUCTION

Sepsis is defined as a life‐threatening organ dysfunction caused by an overactive and extreme response to an infection.^1^ Despite important advances in medical technology in recent years, sepsis still maintains high morbidity and mortality.^2^, ^3^ Approximately 31 million cases of sepsis occur worldwide annually, and the nosocomial fatality rate can reach 30%.^3^, ^4^ During sepsis, the host exhibits overwhelming activation of inflammation induced by infectious pathogens and forms a cytokine storm characterized by the release of abundant proinflammatory factors, including cytokines, chemokines, nitric oxide, histamine, and so on.^5^ An over‐activated proinflammatory response eventually leads to organ dysfunction and even the death of affected individuals.^2^, ^6^ Unfortunately, specific therapy for multiple organ system failure during sepsis is deficient.^7^

On account of the special anatomical location, the liver is most vulnerable to attack by circulating antigenic, bacterial endotoxins, and even pathogens, resulting in the liver being a common target of over‐activated inflammation during infection.^3^, ^8^ Thus, the liver is more vulnerable to damage than any other organ in sepsis. In addition to the damage directly mediated by pathogens, the exaggerated inflammatory response, endothelial injury, microcirculatory failure, and medication side effects may significantly contribute to sepsis‐induced liver injury.^9^ Through pathogen clearance, the synthesis of acute phase reactive proteins, and altered metabolic activity in response to inflammatory processes, the liver exhibits an important role in regulating the immune response to sepsis.^3^, ^10^ The incidence of sepsis‐associated liver failure is difficult to estimate, but as a complication of sepsis, liver failure would undoubtedly worsens patient outcomes.^9^, ^11^, ^12^ Unfortunately, there is a lack of effective treatments to fully restore damaged liver functions. Thus, it is essential to determine the mechanism of liver injury in sepsis to explore therapeutic strategies for liver injury.

Autophagy plays a crucial role in the regulation of inflammation, serving as a mechanism for the clearance of damaged cellular components and the regulation of immune responses. Autophagy can selectively degrade inflammasomes, which are multiprotein complexes involved in the activation of proinflammatory cytokines. It also helps to remove damaged mitochondria and reactive oxygen species, thereby limiting the production of inflammatory mediators. Enhanced autophagy activity contributes to the relief of inflammatory responses.^13^ Autophagy‐related protein 13 (Atg13) is a critical player in the initial phase of autophagy, acting as a key regulator of the recruitment and activation of the ULK1 (unc‐51‐like kinase 1) kinase complex comprising FIP200 (focal adhesion kinase family interacting protein of 200 kDa), ATG13, and ATG101. By promoting the formation and activation of the ULK1 complex, Atg13 enables the proper assembly of the phagophore, thus initiating autophagosome formation.^14^

Long noncoding RNAs (lncRNAs) are a set of RNAs longer than 200 nucleotides that have no coding potential, with the characteristic of low evolutionary conservation of the sequence of the eukaryotic transcriptome.^15^, ^16^ The regulatory mechanisms of lncRNAs vary based on their nuclear or cytoplasmic locations in the cells, and lncRNAs participate in the development of various diseases through regulating genomic imprinting, RNA alternative splicing, and chromatin modification.^17^, ^18^ Many studies have reported that lncRNAs play important roles in liver diseases, such as hepatic fibrosis and liver cancer. The lncRNAs HOTAIR, HULC, and others were reported to be with a high expression level in patients with hepatocellular carcinoma (HCC) or hepatitis B virus and have the potential to become a new diagnostic biomarker.^19^, ^20^, ^21^, ^22^ In contrast, lncRNA MEG3 was found to be downregulated in HCC and liver fibrosis.^18^, ^23^ Whereas, these findings are mainly focused on the functional regulation of lncRNAs in liver fibrosis or HCCs, and lncRNAs in sepsis‐induced liver injury need to be studied systematically.

In this study, by analyzing the library of lncRNAs in liver tissues from endotoxemic mice, we identified a novel lncRNA named lipopolysaccharide (LPS)‐induced liver autophagy regulator (*LILAR*), which regulates autophagy activity through a competing endogenous RNA (ceRNA) mechanism.

## 2 RESULTS

### 2.1 Preparation of the liver injury model of endotoxemic mice

To identify the lncRNAs involved in the liver injury of endotoxemic mice, we prepared an endotoxemic liver injury model in mice by intraperitoneal injection of LPS,^24^ which was followed by a systemic evaluation at the molecular, morphological, and functional levels (Figure S1). The LiquiChip assay results indicated that the serum cytokine levels of the interleukin‐6 (IL‐6), IL‐1β, and tumor necrosis factor (TNF) were markedly increased after LPS treatment for 12 h (Figure S1A). The serum levels of aspartate aminotransferase (AST), lactate dehydrogenase (LDH), and alanine aminotransferase (ALT) were also elevated in mice with injection of LPS for 12 h (Figure S1B). Hematoxylin and eosin (H&E) staining showed that mice challenged with LPS exhibited more extensive histopathological changes in their liver tissue, including infiltration of inflammatory cells, hemorrhage, and hepatocyte necrosis (Figure S1C). Survival analysis showed that LPS injection (20 mg/kg) resulted in high mouse mortality (Figure S1D). These results indicated that the model of LPS‐induced liver injury was established successfully.

### 2.2 Identification of novel lncRNAs

**FIGURE 1:**
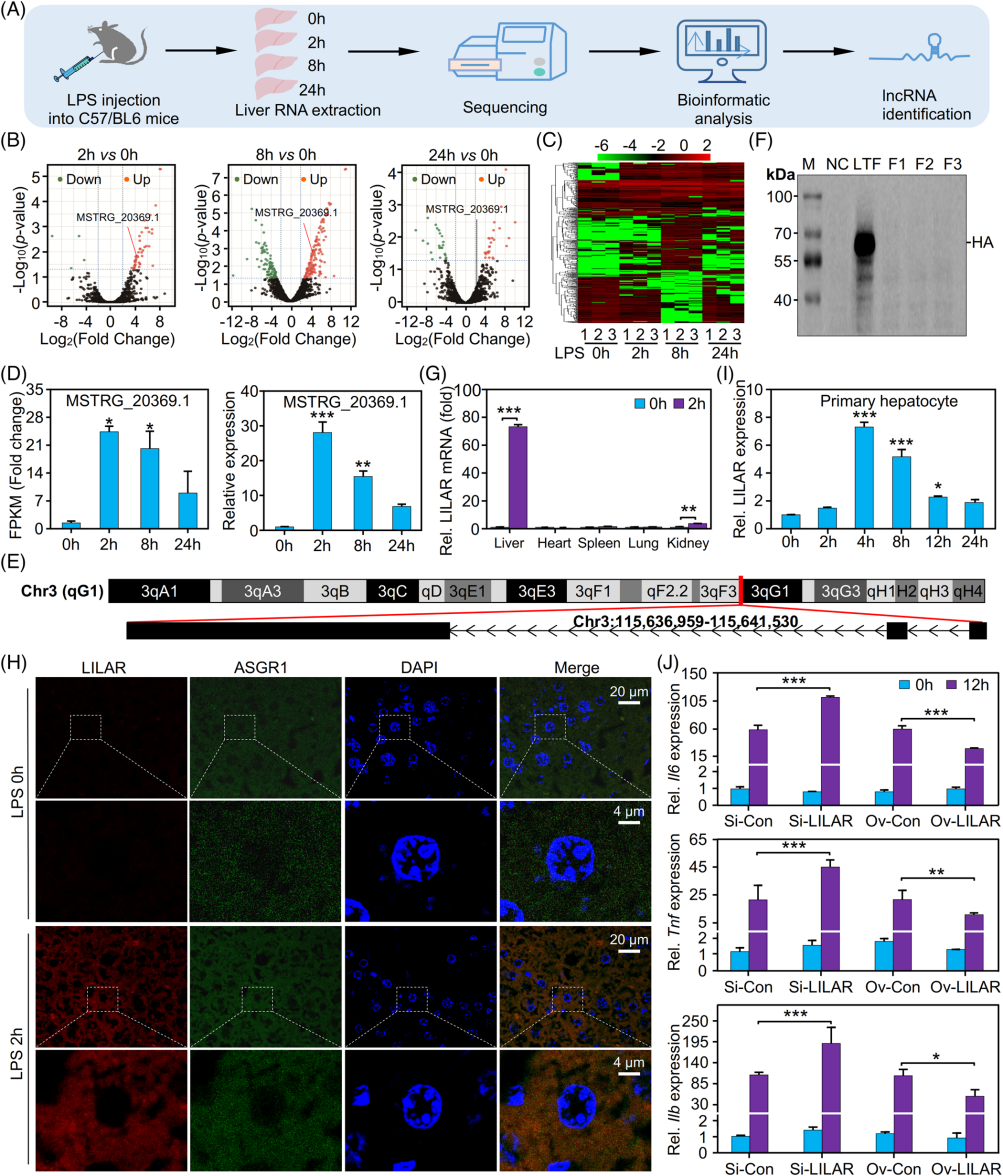
Identification of a novel long noncoding RNA (lncRNA), lipopolysaccharide (LPS)‐induced liver autophagy regulator (*LILAR*), from the liver tissues of mice treated with LPS by RNA‐sequencing (RNA‐seq). (A) Flowchart showing the experimental procedure. Each specific time point group contained three mice. (B) Volcano plots for differentially expressed lncRNAs in the mice treated without or with LPS for different times. Red and green dots represent up‐ and downregulated lncRNAs, respectively. (C) Hierarchical cluster analysis of differentially expressed lncRNAs identified in the liver tissues of mice treated with LPS at different times. The log_10_(FPKM + 0.000001) values were normalized and clustered with a heatmap. Red and green bands represent high and low expressed lncRNAs, respectively. (D) Relative expression of the transcript MSTRG_20369.1 in the liver tissues of mice treated without or with LPS (20 mg/kg, intraperitoneally) at different times. FPKM fold change data of RNA‐seq (left) and quantitative real‐time polymerase chain reaction (qRT‐PCR) results (right). *n* = 3/group. (E) The chromosome location of MSTRG_20369.1 in the mouse genome. (F) Western blot analysis of the coding disability of *LILAR*. Three possible open reading frames (ORFs) of *LILAR* were cloned into the eukaryotic expression vector pcDNA3 with a hemagglutinin (HA) tag. The 293T cells transfected with empty vector pcDNA3 were used as a negative control (NC), while those transfected with a vector expressing HA‐tagged lactotransferrin (LTF‐HA) were used as a positive control. (G) Quantitation of *LILAR* expression in heart, liver, spleen, lung, and kidney tissues of mice treated without or with LPS for 2 h by qRT‐PCR. *n* = 3/group. (H) Fluorescence in situ hybridization (FISH) analysis of *LILAR* expression and location in the liver tissues of mice treated without or with LPS for 2 h. (I) *LILAR* expression in primary hepatocytes treated without or with LPS (1 μg/mL) at different times. *n* = 3/group. (J) Effects of *LILAR* knockdown and overexpression on the expression of *Il6*, *Tnf*, and *Il1b* in primary hepatocytes treated with LPS (1 μg/mL) for 12 h. *n* = 3/group. ^*^ *p* < 0.05; ^**^ *p* < 0.01; ^***^ *p* < 0.001.

To identify novel lncRNAs in the endotoxemic mouse liver tissues, high‐throughput RNA‐sequencing (RNA‐seq) analyses of the livers from mice treated with LPS at indicated times were performed (Figure 1A). Based on Liu et al.’s study,^25^ we determined that the time points of 2, 8, and 24 h would yield valuable insights into the inflammatory response at different stages of endotoxemia, including the initial phase of the response, the transitional phase, and the subsequent recovery phase, respectively. This selection allowed for a comprehensive evaluation of the dynamic changes in lncRNA expression and their potential functional roles during the progression of endotoxemia. We obtained high‐quality RNA‐seq data that met the requirements for bioinformatic analysis (Figures S2 and S3).

Four online software programs, including the Coding Potential Assessment Tool (CPAT), Pfam protein domain analysis, Coding Potential Calculator (CPC), and Coding‐Non‐Coding Index (CNCI), were utilized to predict the protein‐coding potential of these RNA transcripts.^26^, ^27^, ^28^, ^29^ A Venn diagram was plotted to show the lncRNAs identified using these open‐source software programs (Figure S4A). A total of 3056 lncRNA candidates were determined to be putative lncRNAs by the intersection of the four methods. Four types of lncRNAs were obtained: long intergenic noncoding RNAs (lincRNAs) representing 56.3% (1721), intronic lncRNAs representing 11% (337), antisense lncRNAs representing 29.3% (896), and sense lncRNAs representing 3.3% (102) (Figure S4B). Interestingly, we found that most (2360) of the lncRNAs were novel (Figure S4C).

To identify the differentially expressed lncRNAs induced by LPS, we constructed volcano plots with the RNA‐seq data of liver tissues from mice (Figure 1B). In comparison with the control (0 h) group, 81, 433, and 83 RNA transcripts were identified as differentially expressed lncRNAs (|log2(fold change)| > 2; adjusted *p‐*value < 0.05) in livers from mice treated with LPS for 2, 8, or 24 h, respectively (Figure 1B). The differentially expressed lncRNAs were analyzed by hierarchical clustering analysis (Figure 1C).

To illustrate the novel lncRNAs involved in liver dysfunction in sepsis, we focused on the lncRNA MSTRG_20369.1. RNA‐seq data showed that the fragments per kilobase of exon per million mapped fragments (FPKM) of lncRNA MSTRG_20369.1 were significantly increased and reached their peak 2 h after the LPS challenge, which was further confirmed by quantitative real‐time polymerase chain reaction (qRT‐PCR) (Figure 1D and Table S1). The novel lncRNA MSTRG_20369.1 is located in the region 115,636,959–115,641,530 of mouse genome chromosome 3, has a genome span of 4572 bp and a transcript length of 1925 nt and consists of three exons (Figure 1E). The following experiments proved that the lncRNA MSTRG_20369.1 was involved in liver autophagy in endotoxemic mice; therefore, we named this novel lncRNA LPS‐induced liver autophagy regulator (*LILAR*). To verify the coding capability of *LILAR*, we cloned the three potential open reading frames (ORFs), that is, frame 1 (F1), frame 2 (F2), and frame 3 (F3) into the pcDNA3 plasmid vector with a hemagglutinin (HA) tag. After the transfection of the pcDNA3/*LILAR*‐HA recombinant plasmid into 293T cells, the mRNA level of *LILAR* was significantly increased (Figure S5), suggesting that *LILAR* was successfully overexpressed in 293T cells. However, the western blot analysis using a specific HA tag antibody did not show any protein expression for the three ORFs; in contrast, the positive control plasmid pcDNA3/lactotransferrin‐HA was found to have a high level of expression of HA‐tagged lactotransferrin protein (Figure 1F), indicating that *LILAR* lacks the capability to encode proteins, confirming its classification as a lncRNA.

To determine the expression levels of *LILAR* in different organs of mice, we performed qRT‐PCR with lung, heart, spleen, kidney, and liver tissues from mice challenged with or without LPS injection. Intriguingly, the expression of *LILAR* was found to be extremely increased in liver tissue from mice challenged with LPS in comparison with other tissues (Figures 1G and S6), indicating that *LILAR* might have an important function in the liver tissue with a spatiotemporal characteristic. Additionally, we observed a significant increase in the expression level of *LILAR* in the kidney after 2 h of LPS stimulation, suggesting its potential role in the kidney. However, further research is needed to confirm this finding. The spatiotemporal distribution of lncRNA expression has an important influence on its function.^30^ Thus, we performed fluorescence in situ hybridization (FISH) to explore the *LILAR* distribution in mouse liver tissue. FISH analysis demonstrated that *LILAR* was highly increased in the hepatic cells in mouse liver tissues after treatment with LPS for 2 h, displaying a characteristic of cytoplasmic localization (Figures 1H and S7 and Table S2). To further confirm the results above, we performed qRT‐PCR to detect *LILAR* expression in the primary hepatocytes challenged with LPS at indicated times and found that *LILAR* was highly expressed in hepatocytes with a peak at 4 h after the LPS challenge (Figure 1I). In Figure 1D,I, the peak expression time points of *LILAR* appear to be different. This discrepancy may be attributed to variations in the cellular response to LPS due to differences in the in vivo and in vitro environments.

To characterize the biological function of *LILAR* in hepatocytes, we transfected the primary hepatocytes with the plasmid of pcDNA3‐*LILAR* and found that overexpression of *LILAR* dramatically decreased the proinflammatory cytokine levels, such as *Il6*, *Tnf*, and *Il1b* (Figures 1J and S8 and Table S1). On the other hand, we found that *LILAR* knockdown by RNA interference technique markedly upregulated the expression levels of these proinflammatory cytokines in the primary hepatocytes during the treatment with LPS for 12 h (Figures 1J and S8 and Table S2). These results indicated that *LILAR* downregulated LPS‐induced inflammatory response in hepatocytes and might play a protective role in endotoxemia.

### 2.3 *LILAR* deficiency aggravated LPS‐induced liver injury in endotoxemic mice

To further determine the role of *LILAR* in sepsis, we constructed *LILAR* knockout (KO) (*LILAR* ^−/−^) mice by the CRISPR/Cas9 technology (Figure S9).^31^ *LILAR* ^−/−^ mice and wild‐type (WT) littermate controls were subjected to intraperitoneal injection with a lethal dose of LPS (20 mg/kg). In the analysis of mouse survival rate, we observed an increase in mortality 12 h after LPS stimulation, indicating more significant organ damage in mice at this time point (Figure S1). Therefore, we selected this time point to analyze the functional role of *LILAR*.

**FIGURE 2:**
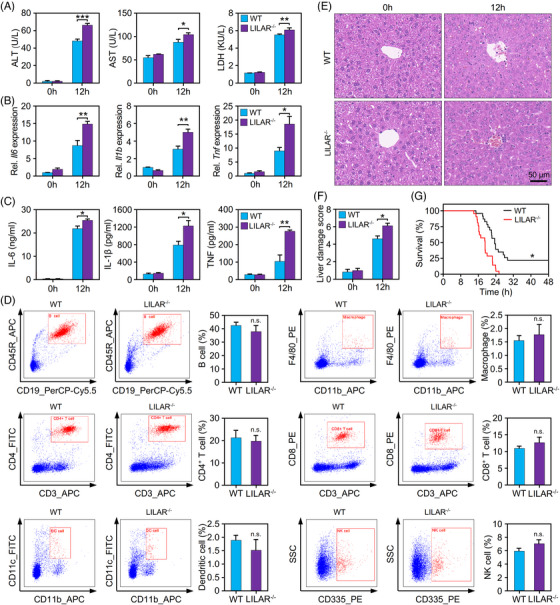
Deficiency of *LILAR* aggravates liver injury in endotoxemic mice induced by lipopolysaccharide (LPS). (A) Quantification of alanine aminotransferase (ALT), aspartate aminotransferase (AST), and lactate dehydrogenase (LDH) in the serum of wild‐type (WT) and *LILAR* knockout (KO) (*LILAR* ^−/−^) mice treated without or with LPS for 12 h. *n* = 3/group. (B) Quantitation of *Il6*, *Il1b*, and *Tnf* mRNA expression in the liver tissues of WT and *LILAR* ^−/−^ mice treated without or with LPS for 12 h by quantitative real‐time polymerase chain reaction (qRT‐PCR). *n* = 3/group. (C) Protein quantitation of interleukin‐6 (IL‐6), IL‐1β, and tumor necrosis factor (TNF) in the serum of WT and *LILAR* ^−/−^ mice treated without or with LPS for 12 h. *n* = 3/group. (D) Influence assessment of *LILAR* deficiency on immune system development. A single‐cell suspension was obtained from the spleens of WT and *LILAR* ^−/−^ mice. Flow cytometric analysis of splenic immune cells was performed with specific antibodies as indicated, and cell populations were gated based on the area and aspect ratio. *n* = 3/group. (E and F) Histopathological examination of livers from WT and *LILAR* ^−/−^ mice treated without or with LPS for 12 h. Hematoxylin and eosin (H&E) staining (E) and liver tissue damage score assessment (F) were performed as described in the methods. *n* = 6/group. (G) Survival curves of WT and *LILAR* ^−/−^ mice (*n* = 20/group) treated with LPS. Statistical analysis was performed using the log‐rank (Mantel–Cox) test. ^*^ *p* < 0.05; ^**^ *p* < 0.01; ^***^ *p* < 0.001; n.s., no significance.

We found that the enzyme activities of ALT, AST, and LDH in serum were significantly elevated in *LILAR^−/−^* mice in comparison with WT mice (Figure 2A). Compared with WT mice, the gene expression levels of proinflammatory cytokines, including *Il6*, *Il1b*, and *Tnf*, in liver tissue were highly increased in *LILAR* ^−/−^ mice (Figure 2B). Consistently, the serum levels of these proinflammatory cytokines were also observably elevated in *LILAR* ^−/−^ mice (Figure 2C). To exclude the possibility that the increase in IL‐6, IL‐1β, and TNF was caused by different compositions of immune cells in *LILAR* ^−/−^ mice, we performed an immunophenotyping analysis of immune cells in spleen tissue (Figure 2D and Table S3). The flow cytometry data showed that there were no significant differences in the innate and adaptive immune cells between WT and *LILAR* ^−/−^ mice, suggesting that *LILAR* deficiency did not significantly influence the development of immune cells. To clarify whether *LILAR* deficiency influenced the biological functions of macrophages, we performed assays for migration and phagocytosis of bone marrow‐derived macrophages (BMDMs) from WT and *LILAR* ^−/−^ mice and found that *LILAR* deficiency failed to induce functional changes of BMDMs from mice (Figure S10). Histopathological examination was performed by H&E staining of liver tissues. In comparison with WT mice, *LILAR* ^−/−^ exhibited more extensive histopathological changes in their liver tissue, including infiltration of inflammatory cells, hemorrhage, and hepatocyte necrosis (Figure 2E,F). After finding that *LILAR* deficiency brings about more severe liver damage, we tested whether *LILAR* ^−/−^ mice subjected to endotoxemia had an increased mortality rate. As predicted, we found that the mortality of *LILAR* ^−/−^ mice treated with LPS was significantly increased compared to that of WT mice (Figure 2G).

Taken together, our results demonstrated that *LILAR* deficiency increased the susceptibility of mice to LPS, which was characterized by elevated cytokines in serum, more severe liver dysfunction, and a reduced survival rate.

### 2.4 *LILAR* overexpression rescued liver injury in *LILAR^−/−^* mice subjected to LPS

**FIGURE 3:**
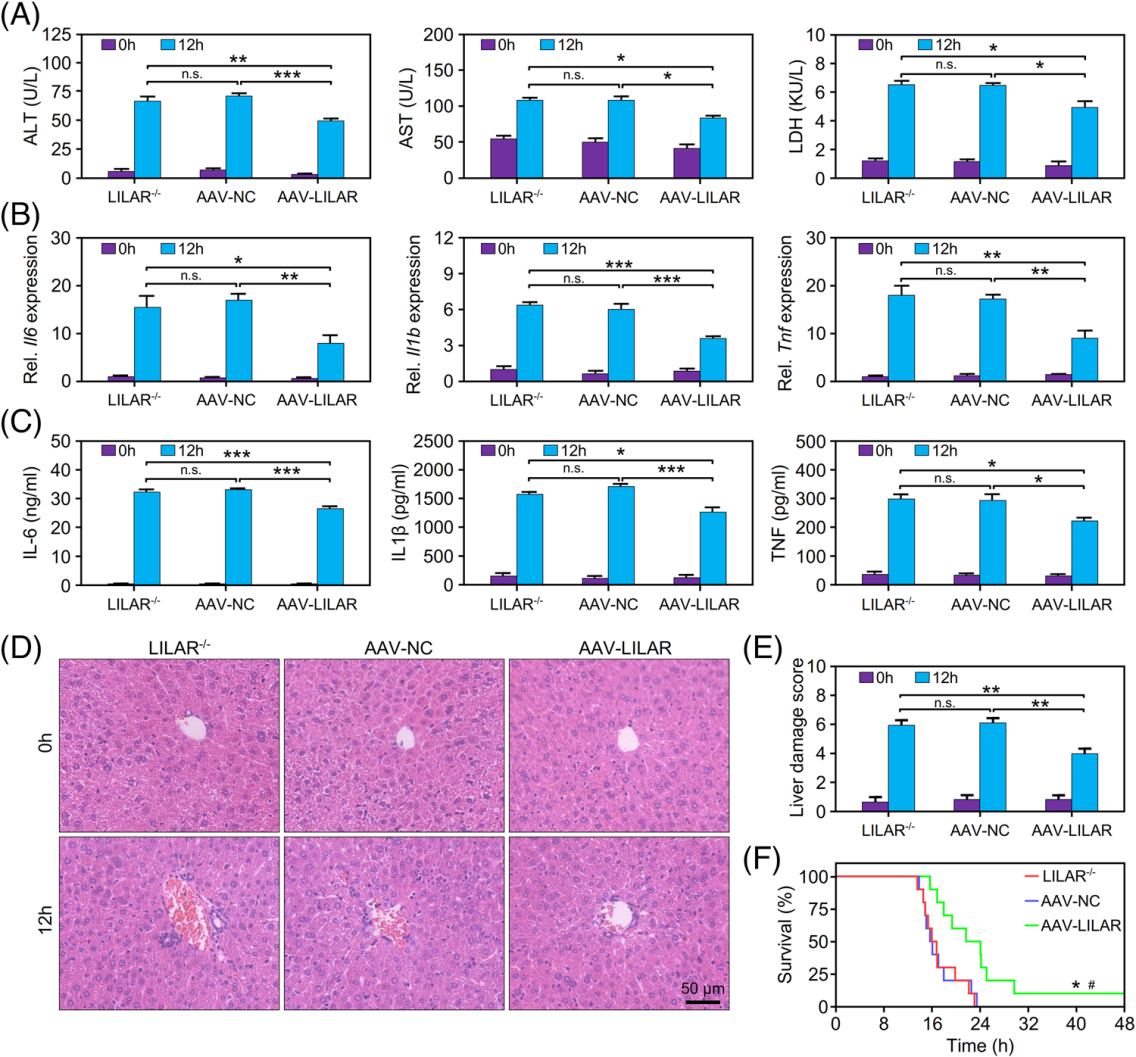
*LILAR* overexpression rescues liver injury in *LILAR‐*deficient mice with endotoxemia. (A) Effect of *LILAR* overexpression by AAV9‐*LILAR* on alanine aminotransferase (ALT), aspartate aminotransferase (AST), and lactate dehydrogenase (LDH) in the serum of *LILAR* ^−/−^ mice challenged without or with lipopolysaccharide (LPS) for 12 h. *n* = 3/group. (B) Effect of *LILAR* overexpression by AAV9‐*LILAR* on *Il6*, *Il1b*, and *Tnf* mRNA expression in the liver tissues of *LILAR* ^−/−^ mice challenged without or with LPS for 12 h. *n* = 3/group. (C) Effect of *LILAR* overexpression by AAV9‐*LILAR* on the protein levels of interleukin‐6 (IL‐6), IL‐1β, and tumor necrosis factor (TNF) in the serum of *LILAR* ^−/−^ mice challenged without or with LPS for 12 h. *n* = 3/group. (D and E) Histopathological examination of livers from AAV9‐*LILAR* pretreated *LILAR* ^−/−^ mice challenged without or with LPS for 12 h. Hematoxylin and eosin (H&E) staining (D) and liver tissue damage score assessment (E) were performed as described in the methods. *n* = 6/group. (F) *LILAR* overexpression by AAV9 rescued the survival rate of *LILAR* ^−/−^ mice challenged with LPS (*n* = 10/group). Statistical analysis was performed using the log‐rank (Mantel–Cox) test. ^*^ *p* < 0.05 compared with *LILAR* ^−/−^ group for survival analysis; ^#^ *p* < 0.05 compared with AAV‐NC group for survival analysis. ^*^ *p* < 0.05; ^**^ *p* < 0.01; ^***^ *p* < 0.001; n.s., no significance.

To confirm the role of *LILAR* in LPS‐induced liver dysfunction, we injected AAV9‐*LILAR* to overexpress *LILAR* in *LILAR* ^−/−^ mice. The qRT‐PCR data showed that *LILAR* was successfully overexpressed in the liver tissues of *LILAR*‐deficient mice (Figure S11). The results showed that *LILAR* expression by AAV9‐*LILAR* significantly reduced the elevated serum levels of ALT, AST, and LDH in *LILAR* ^−/−^ mice subjected to LPS for 12 h in comparison with the AAV control (AAV‐NC) (Figure 3A). In addition, the expression levels of proinflammatory cytokines at the mRNA level including *Il6*, *Il1b*, and *Tnf* in liver tissues were downregulated by AAV9‐*LILAR* injection in *LILAR* ^−/−^ mice challenged with LPS (Figure 3B) in comparison with the AAV control. In line with this result, the overexpression of *LILAR* also reduced the elevated serum cytokine levels (IL‐6, IL‐1β, and TNF) of *LILAR* ^−/−^ mice treated with LPS for 12 h (Figure 3C).

Histopathological examination was performed with H&E staining to evaluate liver tissue injury, and we found that *LILAR* overexpression by AAV9‐*LILAR* significantly rescued the histopathological changes in the liver tissue, including the hepatic sinus congestion, neutrophil infiltration, and hepatic cord structure destruction, in *LILAR* ^−/−^ mice at 12 h after LPS injection (Figure 3D,E). Based on the findings above, we tested whether AAV9‐mediated *LILAR* overexpression influenced the survival of *LILAR* ^−/−^ mice with the injection of LPS. The results illustrated that *LILAR* overexpression by AAV9 markedly improved the survival rate of *LILAR* ^−/−^ mice treated with intraperitoneal injection of LPS compared with the AAV control group (Figure 3F).

Taken together, our results demonstrated that *LILAR* overexpression significantly rescued *LILAR*‐deficiency‐mediated liver tissue injury in mice with endotoxemia, thus increasing the survival of *LILAR* ^−/−^ mice.

### 2.5 *LILAR* deficiency reduced autophagy in the liver by downregulating Atg13 expression

The identification of target genes regulated by *LILAR* is crucial for comprehending the underlying mechanisms of its biological function in endotoxemia. In the animal model, *LILAR* reaches its peak expression level at 2 h. Considering that the manifestation of its biological effects may have a delayed onset, we conducted transcriptome sequencing analysis on liver tissue samples obtained after 4 h, both with and without LPS treatment. The quality of RNA‐seq data met the requirements for subsequent bioinformatic analysis (Figure S12), and the Pearson correlation coefficients between intragroup samples showed high reproducibility for the biological replicates (Figure S13).

**FIGURE 4:**
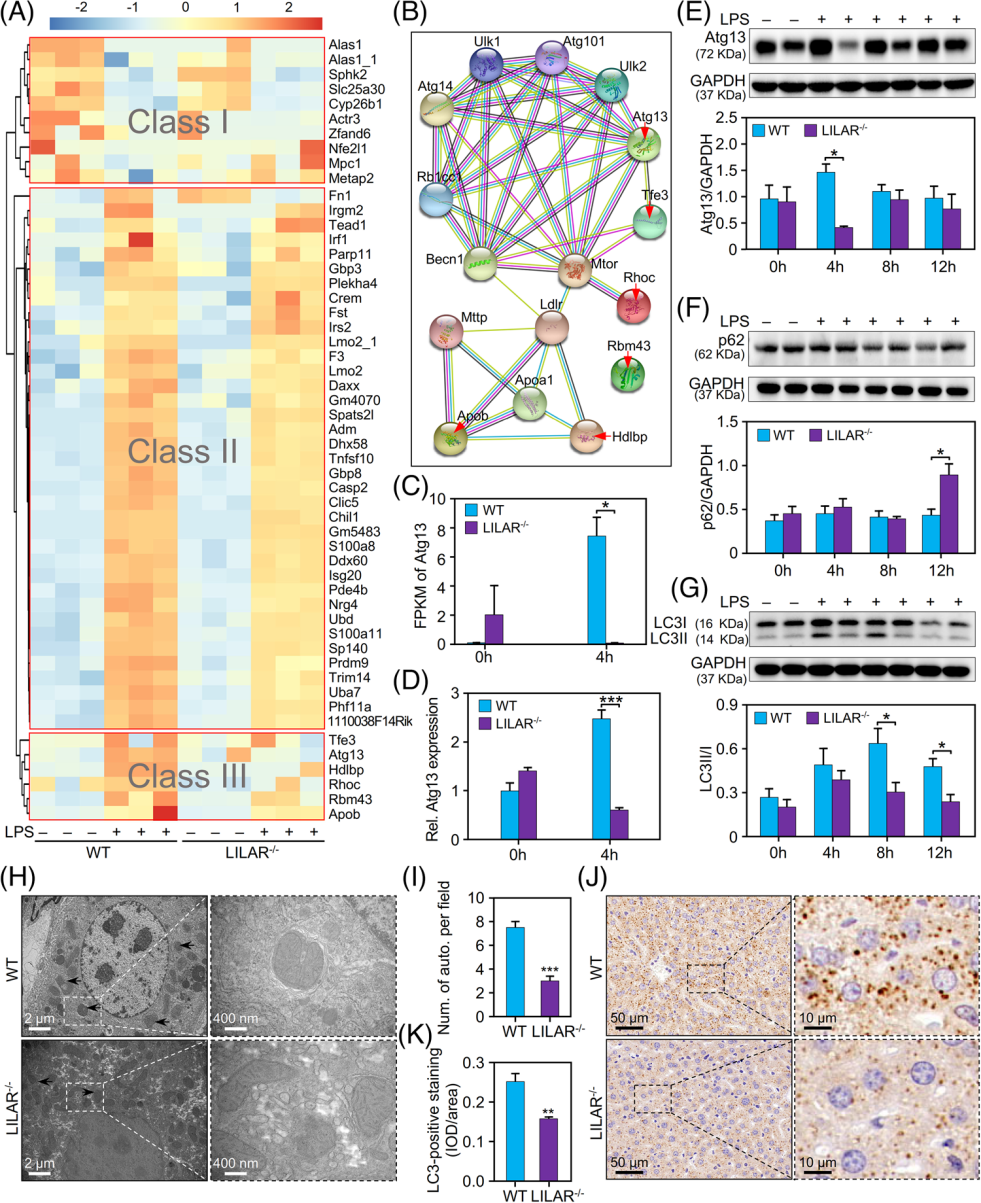
*LILAR* deficiency reduces lipopolysaccharide (LPS)‐induced autophagy in liver tissues by downregulating autophagy‐related protein 13 (Atg13) expression. RNA‐sequencing (RNA‐seq) analysis was performed using livers from wild‐type (WT) and knockout (KO) (*LILAR* ^−/−^) mice (*n* = 3/group) treated without or with LPS for 4 h. (A) Heatmap and clustering analysis of the differentially expressed genes. All the differentially expressed genes (DEGs) were clustered into Classes I, II, and III. (B) STRING analysis of gene products from Class III by setting the first shell with no more than 10 interactors. The genes from Class III are indicated by red arrows. (C) Quantitation of Atg13 mRNA by FPKM values from RNA‐seq data. (D) Quantitation of Atg13 mRNA expression by quantitative real‐time polymerase chain reaction (qRT‐PCR). *n* = 3/group. (E–G) Western blot analysis of Atg13 (E), p62 (F), and LC3B (G) protein expression in the liver tissues of WT and *LILAR* ^−/−^ mice treated without or with LPS (20 mg/kg) for 4, 8, and 12 h. *n* ≥ 3/group. (H) Electron microscopy examination of autophagy structures in liver tissues from WT and *LILAR* ^−/−^ mice treated with LPS for 12 h. The autophagosome was indicated by the black arrow. (I) The histogram displaying the number of autophagosomes per field. *n* = 4/group. (J) Immunohistochemistry staining of LC3B in the liver tissues from WT mice and *LILAR* ^−/−^ mice treated with LPS for 12 h. (K) Quantitative analysis of the level of LC3B by immunohistochemical staining (*n* = 6/group). IOD, integrated optical density. ^*^ *p* < 0.05; ^**^ *p* < 0.01; ^***^ *p* < 0.001.

Volcano plots showed that there were 2831 and 2683 differentially expressed genes (DEGs) (|log_2_(fold change)| > 1; adjusted *p‐*value < 0.05) in the WT and *LILAR^−/−^* mice treated with LPS for 4 h, respectively (Figure S14). To further determine the DEGs regulated by *LILAR* in response to LPS, the genes with extremely low expression with fewer than five total reads in each group were removed. The *LILAR‐*regulated genes were screened out by the standard that the ratio of gene induction between the *LILAR* ^−/−^ and WT groups [(FPKM*_LILAR_*_‐KO4h_ – FPKM*_LILAR_*_‐KO0h_)/(FPKM_WT4h_ – FPKM_WT0h_)] was >2 or <0.5 and the relative fold change in gene expression of *LILAR* ^−/−^ mice in comparison with WT mice [(FPKM*_LILAR_*_‐KO4h_/FPKM *_LILAR_*_‐KO0h_)/(FPKM_WT4h_/FPKM_WT0h_)] was >2 or <0.5. Finally, we obtained 53 DEGs regulated by *LILAR* in response to LPS. These *LILAR‐*regulated DEGs were visualized in a heatmap and clustered into three classes according to their expression profiles named Class I, Class II, and Class III, highlighted with larger gray font in Figure 4A. To understand the functional interactions among these gene products, we performed STRING protein–protein interaction analysis with the products of each class of genes.^32^ Interestingly, there were no strong interactions in Class I and Class II genes (Figure S15). Notably, all the proteins coded by Class III genes, except Rbm43, formed a strong interaction network closely associated with autophagy, suggesting that *LILAR* might regulate the autophagy activity of liver tissue from mice challenged with LPS (Figure 4B).

Atg13 is an autophagy factor required for autophagosome formation and mitophagy.^14^ RNA‐seq data showed that the FPKM of the Atg13 transcript was significantly increased in WT mice after LPS injection for 4 h but was inversely downregulated in *LILAR* ^−/−^ mice subjected to LPS treatment (Figure 4C). Convincingly, this result was verified by qRT‐PCR with liver tissues from WT or *LILAR* ^−/−^ mice (Figure 4D and Table S1). In line with the results of gene expression, Atg13 protein was also found to be decreased in *LILAR* ^−/−^ mice in comparison with WT mice (Figure 4E). Thus, we speculated that *LILAR* may be involved in the autophagy regulation of liver tissue in endotoxemic mice through Atg13. To validate our hypothesis, we detected the autophagy‐related protein expression levels, such as LC3B and p62, in the liver tissues of mice subjected to LPS (Figure 4F,G). LC3B is a well‐established marker for monitoring autophagy activity. LC3B‐I is the cytosolic form of LC3B, while LC3B‐II is the result of LC3B‐I lipidation and recruitment to autophagosomal membranes.^33^ The ratio of LC3B‐II/I reflects the proportion of LC3B that has undergone this conversion and is indicative of autophagosome formation. The ratio of LC3‐II/I was downregulated in *LILAR* ^−/−^ mice compared with WT mice after injection of LPS for 8 and 12 h (Figure 4G), which represents the loss of autophagosome formation.^34^ Consistently, we found that p62 protein was significantly increased in *LILAR* ^−/−^ mice compared with WT mice at 12 h after LPS injection induced by the reduction of autophagy activity (Figure 4F). The alterations in p62 protein expression are a consequence of the gradual accumulation of autophagic activity. In *LILAR* KO mice, the progressive decline in autophagic activity gradually leads to an increase in p62. Finally, after 12 h of LPS stimulation, significant differences in p62 expression are observed between WT and *LILAR* KO groups. Furthermore, we performed electron microscopy to observe the formation of autophagosomes and found that, in comparison with WT mice, autophagosome formation was suppressed in the liver tissue of *LILAR* ^−/−^ mice (Figure 4H,I). The LC3B protein has been reported to have a membrane location of autophagosomes.^35^ Thus, we performed immunohistochemistry to detect LC3B and found that, in comparison with WT mice, the LC3B expression level was significantly reduced in the *LILAR* ^−/−^ mouse liver tissues at 12 h after LPS injection (Figure 4J,K). All the data demonstrated that autophagy activity was decreased in *LILAR* ^−/−^ mice by downregulating Atg13 expression, resulting in aggravated liver injury.

### 2.6 *LILAR* regulated the expression of Atg13 through competitive adsorption of miR‐705

**FIGURE 5:**
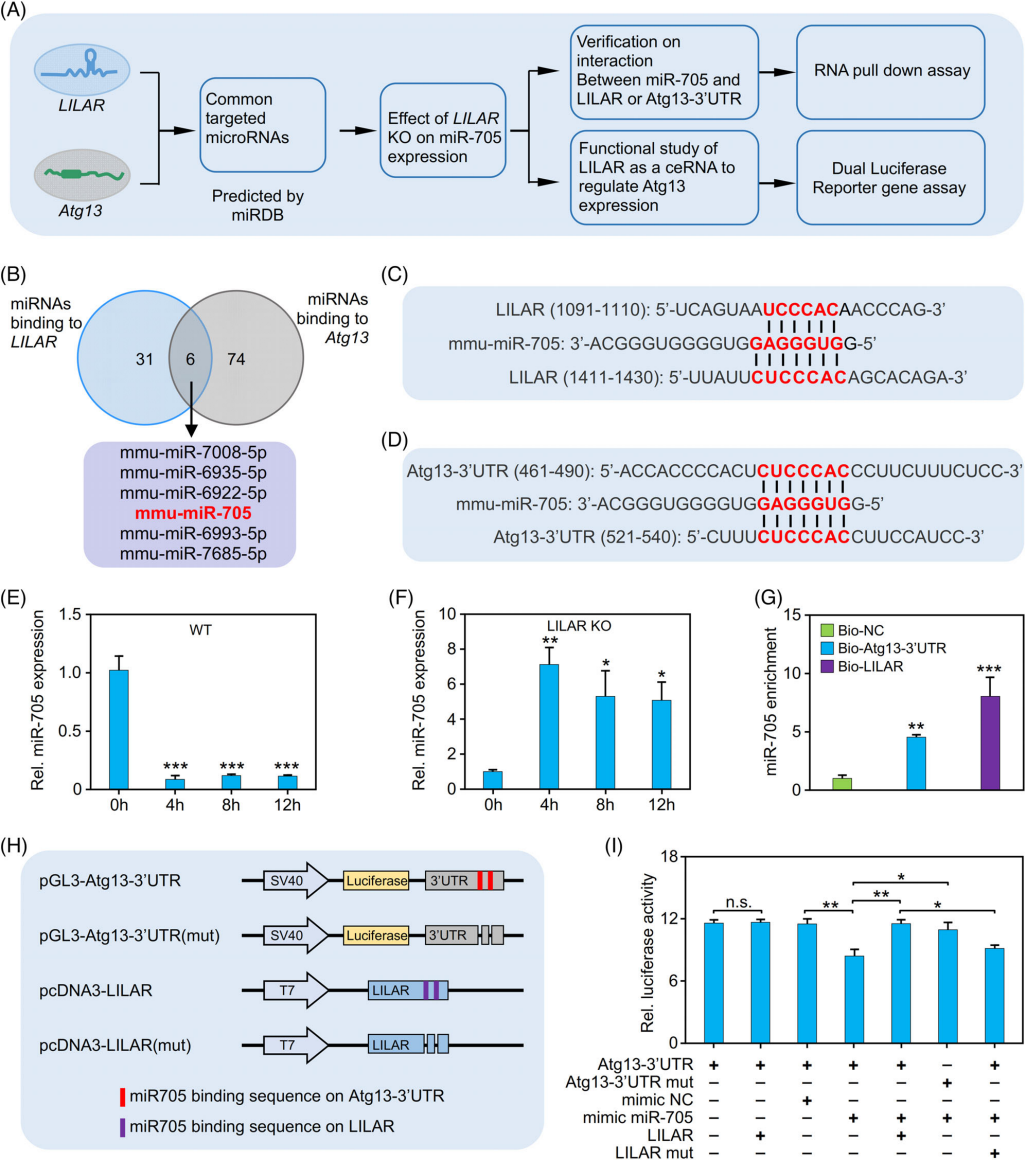
*LILAR* decreases lipopolysaccharide (LPS)‐induced autophagy‐related protein 13 (Atg13) expression in liver cells through competitive binding with miR‐705. (A) An integrated approach to identify the mechanism by which *LILAR* regulates ATG13 in liver cells. (B) Venn diagram of miRNAs binding to *LILAR* or the 3′‐untranslated region (UTR) of *Atg13* predicted by miRDB. (C and D) The predicted miR‐705 binding sites (red text) on *LILAR* (C) and the 3′UTR of Atg13 (Atg13‐3′UTR) (D). (E and F) Quantitative real‐time polymerase chain reaction (qRT‐PCR) quantitation of miR‐705 expression in wild‐type (WT) (E) and knockdown (KO) (F) mice treated without or with LPS for 4, 8, and 12 h. *n* = 3/group. (G) RNA pulldown assay to validate the interaction of miR‐705 with *LILAR* or Atg13‐3′UTR. After RNA pulldown with biotinylated probes for *LILAR* or Atg13‐3′UTR, qRT‐PCR was performed to detect miR‐705 in the pulldown mixture. *n* = 3/group. (H) Schematic construction of pGL3 vectors carrying WT Atg13‐3′UTR or with miR‐705 binding site deletion and pcDNA3 vectors containing WT *LILAR* or with miR‐705 binding site deletion. Predicted binding sites of miR‐705 are illustrated in red for Atg13‐3′UTR and in purple for *LILAR*. (I) Dual‐luciferase reporter gene assay showing *LILAR* competitive binding with miR‐705 to increase Atg13 expression. *n* = 3/group. ^*^ *p* < 0.05; ^**^ *p* < 0.01; ^***^ *p* < 0.001; n.s., no significance.

The ceRNA mechanism is a regulatory pathway that encompasses the interaction between different RNA molecules, such as mRNAs, lncRNAs, and microRNAs (miRNAs). This mechanism functions by facilitating competitive binding of these noncoding RNA molecules to miRNAs, utilizing shared miRNA response elements.^36^ This interaction ultimately leads to the modulation of target gene expression. The ceRNA mechanism is a common mechanism by which lncRNAs exert their regulatory functions. In order to investigate the molecular mechanism underlying the regulation of Atg13 by *LILAR*, we utilized an integrative approach to uncover how *LILAR* modulates Atg13 in the liver tissues of endotoxemic mice (Figure 5A). To test whether the lncRNA *LILAR*‐regulated Atg13 mRNA transcription through a ceRNA mechanism, we utilized the miRDB database (http://mirdb.org/index.html) to predict the miRNAs that interact with *LILAR* and/or the 3′‐untranslated region (UTR) of Atg13 (Atg13‐3′UTR) and found that six miRNAs have the capability to interact with both *LILAR* and Atg13‐3′UTR (Figure 5B).

Of the six miRNAs, miR‐705 has been found to be upregulated in the process of alcohol‐related liver disease (ALD) and associated with autophagy in ALD.^37^, ^38^ In this study, *LILAR* played an important role in regulating hepatic autophagy in LPS‐induced endotoxemia in mice; hence, we speculated that miR‐705 was likely to be a downstream target of *LILAR*. DNA sequence analysis demonstrated that both *LILAR* and Atg13‐3′UTR contain two miR‐705‐binding sites (Figure 5C,D), indicating that *LILAR* might regulate autophagy through competitive binding with miR‐705. The qRT‐PCR results showed that the expression of miR‐705 was significantly decreased in the liver tissues of WT mice after LPS injection for 4−12 h (Figure 5E) but was upregulated in *LILAR* ^−/−^ mice after LPS challenge (Figure 5F). Importantly, the RNA pulldown assay showed that biotinylated probes for both *LILAR* and Atg13‐3′UTR specifically precipitated miR‐705 in vitro (Figure 5G).

To further identify the functional regulation effect of *LILAR* on Atg13 expression, we constructed a dual‐luciferase reporter gene system, including pGL3/Atg13‐3′UTR and pGL3/Atg13‐3′UTR (mut) with deleted binding sites of miR‐705. In addition, we cloned full‐length WT *LILAR* and the mutant with the deletion of miR‐705 binding sites into pcDNA3 (Figure 5H) to test whether *LILAR* competitively adsorbs miR‐705 to reduce its binding to Atg13‐3′UTR and thus to promote the stability of Atg13 mRNA, resulting in the upregulation of Atg13 protein. The results indicated that the transfection of the mimic miR‐705 decreased the transcriptional activity of Atg13 in comparison with the negative control, and *LILAR* overexpression reversed the reduction in Atg13 transactivity mediated by the binding of miR‐705 (Figure 5I). Furthermore, when the miR‐705 binding sites in either *LILAR* or Atg13‐3′UTR were deleted, the phenomenon described above disappeared (Figure 5I).

Based on these results, we conclude that *LILAR* absorbs miR‐705 acting as a molecular sponge, thus decreasing the binding of miR‐705 to Atg13‐3′UTR and resulting in Atg13 mRNA and protein expression.

## 3 DISCUSSION

A growing amount of evidence demonstrates that lncRNAs may have critical regulatory functions in diverse pathological processes or diseases, including inflammation, cancer, autoimmune diseases, and sepsis.^39^, ^40^, ^41^, ^42^ Several lncRNAs, including LINC00472, nuclear enriched abundant transcript 1 (NEAT1), colorectal neoplasia differentially expressed, and X‐inactive‐specific transcript (XIST), have been reported to participate in liver damage induced by sepsis through modulating the inflammatory response.^5^, ^7^ For example, NEAT1 was reported to promote the inflammatory response by regulating the Let‐7a/TLR4 axis in sepsis‐induced liver injury,^43^ whereas silencing the lncRNA XIST protected against sepsis‐induced acute liver injury through the inhibition of bromodomain‐containing protein 4 expression.^44^ However, more work is needed to prove whether lncRNAs exert functional regulation on other aspects, such as autophagy, in sepsis‐induced liver injury.

In order to understand the dynamic changes and functional roles of specific molecules during sepsis progression, it is crucial to select appropriate time points for analysis. In our study, we chose the time points of 0, 2, 8, and 24 h after LPS stimulation in mice based on a previous study conducted by Liu et al. in our research group.^25^ They measured the levels of cytokines in the peripheral blood serum and various organs of LPS‐stimulated mice and found that certain cytokines, such as TNF, peaked at 2 h after LPS stimulation, while other cytokines, such as IL‐6, IL‐10, and IL‐12, gradually started to decline between 6 and 12 h and most cytokines returned to baseline levels by 24 h. Based on these findings, we believed that the time points of 2, 8, and 24 h would provide valuable insights into the inflammatory response of mice at different stages of endotoxemia. These time points capture the early phase response, the transition phase, and the recovery phase, respectively, allowing us to comprehensively analyze the dynamic changes in lncRNA expression and their potential functional roles during sepsis progression. It has been reported that liver dysfunction in patients often occurs in early sepsis, usually on the day of sepsis diagnosis (<24 h after the onset of the disease), because of inflammation and hypoperfusion.^6^, ^10^ Therefore, it is critical for us to understand the roles of lncRNAs in the early stage of sepsis and illustrate the mechanism of sepsis‐induced liver injury. So in this research, we focused on the differentially expressed lncRNAs that were significantly induced by LPS in the early stage of endotoxemia, resulting in the identification of *LILAR*, a novel LPS‐induced lncRNA.

To illustrate the role of *LILAR* in the liver of endotoxemic mice, the CRISPR/Cas9 system was utilized for the construction of *LILAR* KO mice. We found that upon challenge with LPS, the *LILAR* ^−/−^ mice exhibited more extensive inflammation and more severe liver injury than WT mice (Figure 2). Intriguingly, *LILAR* overexpression by AAV9 rescued these pathological changes in the *LILAR* ^−/−^ mouse liver tissues and significantly increased the survival of *LILAR* ^−/−^ mice with endotoxemia (Figure 3). All these results demonstrate that *LILAR* plays a protective role in the liver tissues of endotoxemic mice. To clarify the mechanism by which *LILAR* exerts biological functions, we performed RNA‐seq analysis of liver tissues from *LILAR* ^−/−^ and WT mice subjected to LPS treatment. Hierarchical clustering of RNA‐seq data demonstrated that the genes regulated by *LILAR* in response to LPS were clustered into three classes, that is, Class I, Class II, and Class III (Figure 4A). To explore the potential interaction of the proteins, STRING analysis of the proteins encoded by each cluster of genes was performed and we found that the proteins encoded by genes in Class III were strongly connected with autophagy. Based on the results of bioinformatics analysis, we propose that *LILAR* exhibits a critical role in regulating autophagy activity in the liver tissues of mice. It is well established that autophagy activity protects liver functions in various diseases.^45^ Gene profile analysis of Class III showed that the gene Atg13, which encodes a key element of the ULK1 complex for autophagosome formation,^46^, ^47^ was decreased in *LILAR* ^−/−^ mice treated with LPS, indicating that Atg13 was a regulator of autophagy activity in the *LILAR* ^−/−^ mouse liver tissues. Consistent with the results of gene profile analysis, the qRT‐PCR and western blot experiments exhibited that the mRNA and protein levels of Atg13 were downregulated simultaneously. Interestingly, our findings revealed a significant decrease in the protein ratio of LC3B‐II/I and a significant increase in the protein level of p62 in *LILAR* ^−/−^ mice following LPS treatment. In parallel, the results of electron microscopy and LC3II immunohistochemical analysis also showed a decrease in autophagy activity in the *LILAR* ^−/−^ mouse liver tissues. All these results demonstrate that the lncRNA *LILAR* regulates the autophagy activity in liver tissue during sepsis. Autophagy plays a crucial role in inflammation, where moderate autophagy is beneficial, but excessive autophagy can be detrimental to the organism.^45^, ^48^ In the early stages of inflammation, the body activates autophagy as a protective mechanism. As mentioned in our study, we observed a significant upregulation of Atg13 at 4 h, which is consistent with the activation of autophagy during this early phase. However, as the inflammatory response progresses, there is a need to prevent excessive autophagy‐induced damage to the organism. Therefore, the downregulation of Atg13 observed at 8 h was viewed as a regulatory response by the body to inhibit further autophagic activity. This is supported by the observation that the level of LC3B protein follows a similar pattern to that of Atg13, providing further evidence for our discovery (Figure 4G). Atg13 exhibits a heterogeneous expression pattern in *LILAR* ^−/−^ mice, indicating that the body may employ a compensatory mechanism to enhance autophagy activity as a protective response.

It is very important to explore the role of *LILAR* in modulating Atg13 expression in sepsis. Competitive binding with endogenous RNA is a common mechanism by which lncRNAs affect downstream mRNA expression.^49^, ^50^ For example, Wang et al.^51^ found that a lncRNA, named HULC (a lncRNA highly upregulated in liver cancer), may downregulate miR‐372 as an endogenous “sponge” to improve the translation of protein kinase cAMP‐activated catalytic subunit beta (PRKACB). Recently, Zheng et al.^52^ reported that the nucleotide oligomerization domain 1 (NOD1) antibacterial and antiviral‐related lncRNA (NARL) functions as a ceRNA for miR‐217‐5p to downregulate NOD1 protein in teleost fish, thus controlling the intensity of immune responses. Bioinformatics analysis demonstrated that several miRNAs have a binding activity to Atg13‐3′UTR as well as *LILAR*, of which miR‐705 had been reported to be upregulated with the suppression of autophagy activity in ALD.^37^, ^38^ Interestingly, our results showed that autophagy activity was increased along with the downregulation of miR‐705 expression in liver tissues from WT mice subjected to LPS, whereas in *LILAR* ^−/−^ mice, miR‐705 was upregulated, resulting in a significant decrease in autophagy activity in liver tissues. RNA pulldown assays showed that biotinylated probes for both *LILAR* and Atg13‐3′UTR could specifically precipitate miR‐705 from liver tissue samples, which provides physical evidence for competitive binding of miR‐705 with *LILAR* and Atg13‐3′UTR. Further luciferase assays proved that *LILAR* competitively adsorbed miRNA‐705 to regulate the expression of Atg13 in cultured cells. Based on the results above, we conclude that *LILAR* acts as a ceRNA for miR‐705 to upregulate the expression of Atg13, thus enhancing autophagy activity in liver tissues. To determine the upstream signaling pathway that regulates *LILAR* expression, we detected the expression of *LILAR* in *TLR2^−/−^*, *TLR4^−/−^*, *MyD88^−/−^*, and *TRIF^−/−^* gene KO mice treated with LPS for 2 h (Figure S16), and we found that *TLR4* or *MyD88* gene deficiency, but not *TLR2* and *TRIF* gene deficiency, significantly disrupted the *LILAR* expression induced by LPS, indicating that the *TLR4*‐*MyD88* signaling pathway participated in regulating *LILAR* expression in the liver tissues of mice.

Despite the limited conservation of sequences in lncRNAs, there is evidence of functional conservation among them.^53^ In order to check the conservation of *LILAR*, we performed sequence alignments and identified a transcript in the human transcriptome named ENSG00000283662. This transcript is a lncRNA and exhibits certain sequence similarities with *LILAR*, but the similarity is not very high (Figure S17). Previous studies have demonstrated that low‐conserved sequences of lncRNAs in different species can perform the same functions. For example, Karner et al.^54^ discovered that despite the presence of certain sequence variations between humans and mice, the lncRNA JPX retains its ability to perform the same function by interacting with CTCF (CCCTC‐binding factor) to activate XIST. Further bioinformatics analysis revealed that ENSG00000283662 is capable of interacting with multiple miRNAs, and several of them can interact with the 3′UTR of human ATG13, including hsa‐miR‐665, hsa‐miR‐4640‐5p, and hsa‐miR‐3667‐3p. This result indicates that ENSG00000283662 is a corresponding lncRNA in humans of mouse *LILAR*, which plays comparable roles in the regulation of autophagy. However, further experimental validation is required to confirm these findings.

**FIGURE 6:**
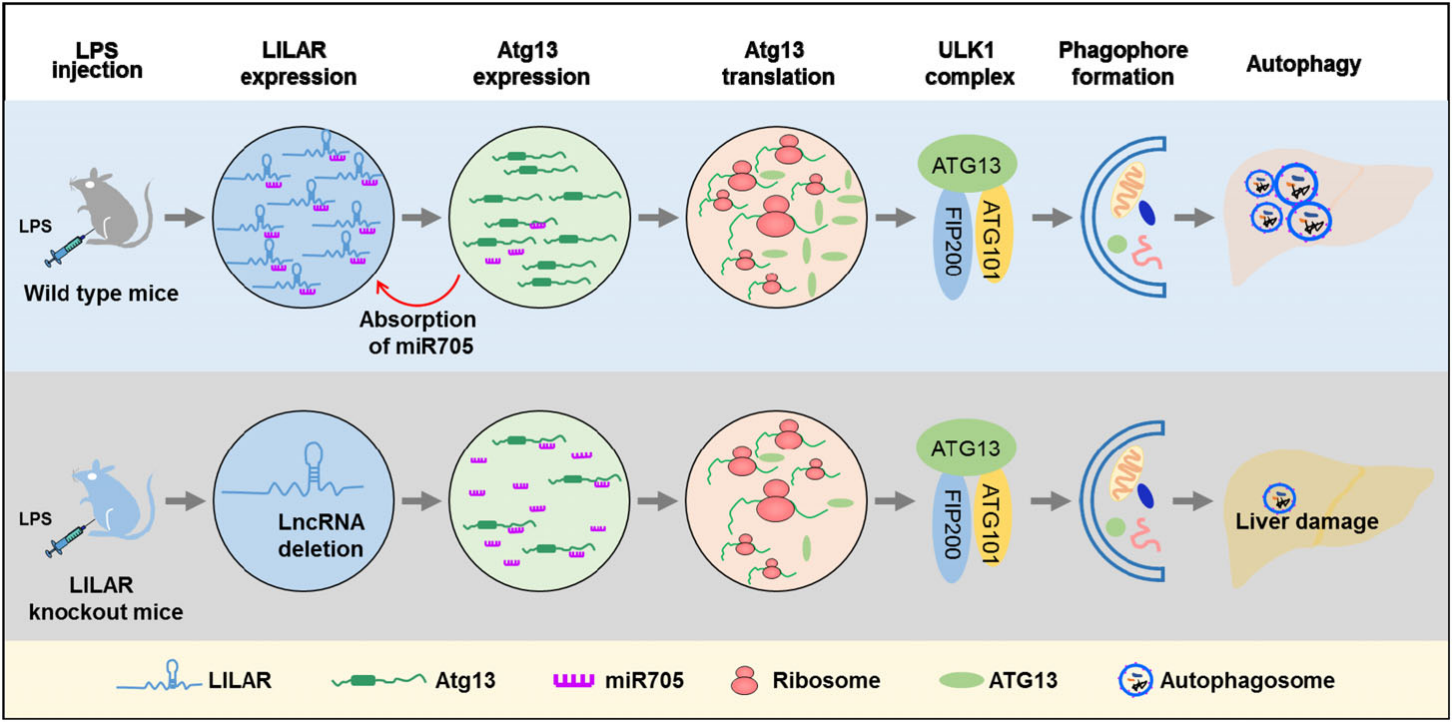
A proposed work model for *LILAR* regulating autophagy in the liver tissues of endotoxemic mice. Upon challenge with lipopolysaccharide (LPS), the expression level of a novel long noncoding RNA, *LILAR*, was increased in the liver tissues of mice. *LILAR* functions as a competing endogenous RNA (ceRNA) that sponges miR‐705 to upregulate the expression of autophagy‐related protein 13 (Atg13) by removing the suppressive miR‐705 bound to its 3′‐untranslated region (UTR). Increased expression of the Atg13 protein promotes the formation of the ULK1 (unc‐51‐like kinase 1) complex comprising FIP200, ATG13, and ATG101, which is necessary for phagophore formation and autophagy activity, resulting in a reduction in liver injury in mice subjected to LPS injection. *LILAR* deficiency downregulates Atg13 through a ceRNA mechanism and suppresses the autophagy activity of liver tissues induced by LPS, leading to an increased death rate in mice. The schematic was drawn with the help of Microsoft PowerPoint.

In conclusion, we finally identified a novel lncRNA, *LILAR*, as the regulator of hepatic autophagy, which was induced in mice in the early phase of endotoxemia. We also provided evidence to show that the *LILAR*–miR‐705–Atg13 axis participated in regulating autophagy in the liver tissues of mice subjected to LPS through a ceRNA mechanism (Figure 6). These *LILAR* findings are not only helpful for understanding the pathophysiology of sepsis‐induced liver injury but also beneficial to the advancement of novel therapeutic approaches to restore the physiological function of damaged liver tissues induced by endotoxemia.

## 4 MATERIALS AND METHODS

### 4.1 Animals

All male C57BL/6 WT mice aged 8–12 weeks were obtained from Southern Medical University. The generation of *LILAR^−/−^* mice was outsourced to Cyagen Biosciences Inc. *TLR2^−/−^*, *TLR4^−/−^*, *MyD88^−/−^*, and *TRIF^−/−^* mice were from Professor T.R. Billiar (University of Pittsburgh). All mice used in the experiments were raised in specific pathogen‐free conditions with free access to water and diet. Mice were anesthetized with isoflurane inhalation. After treatment with LPS (20 mg/kg body weight) at different times, the mice were killed at predetermined times.

### 4.2 Primary hepatocyte isolation and culture

The primary hepatocytes were isolated according to previous reports.^55^ Briefly, after anesthesia, the blood of the liver was washed out with chelate calcium by cannulation via the vena cava. Then, the extracellular matrix was dissociated by perfusion with liberase (Sigma, #05401127001). The liver was dissected for hepatocyte purification via density‐based separation. Finally, hepatocytes were resuspended with fetal bovine serum (FBS) ‐free Roswell Park Memorial Institute (RPMI) 1640 (Gibco, #11875093) and seeded in the plates coated with collagen (Sigma, #C3867‐1VL).

### 4.3 lncRNA analysis

To remove low‐quality data reads and adaptor sequences, Trimmomatic software (v0.38) was used to trim all the raw data.^56^ Next, the sequencing reads were aligned independently to the reference genome of Mus musculus (GRCm39/mm39) using HISAT2 software (v2.1.0).^57^ After mapping the reads to the reference genome, we used StringTie (v1.3.6) to assemble the transcriptome. And the Gffcompare program was performed to annotate the assembled transcripts. We used those transcripts that were not annotated to screen for supposed lncRNAs. Furthermore, we combined four approaches, including CNCI (http://www.bioinfo.org/software/cnci), CPC (http://cpc2.gao‐lab.org/index.php), Pfam database (http://pfam.xfam.org/), and CPAT (http://lilab.research.bcm.edu/cpat/index.php), to screen for the potential lncRNAs in the unannotated transcripts. We selected transcripts that were greater than 200 nt and contained more than two exons as lncRNA candidates. By using cuffcompare (http://cole‐trapnell‐lab.github.io/cufflinks/cuffcompare/), four types of lncRNAs were identified, including lincRNAs, intronic lncRNAs, sense lncRNAs, and antisense lncRNAs.

### 4.4 Differential expression analysis and hierarchical clustering

The standard that adjusted *p‐*value < 0.05 and |log_2_(fold change)| > 2.0 was applied to determine the DEGs for lncRNAs by DEseq2 R package. Hierarchical cluster analysis of differentially expressed lncRNAs in liver tissues was performed with the R package pheatmap (https://rdocumentation.org/packages/pheatmap/versions/1.0.12).

### 4.5 RNA fluorescent in situ hybridization

The expression and subcellular localization of *LILAR* in mouse liver tissues were examined with a FISH Kit from GenePharma following the manufacturer's instructions. The Cy3‐labeled FISH probes are listed in Table S2. Hepatocytes in the liver tissues were labeled with a CoraLite 488‐conjugated asialoglycoprotein receptor 1 (ASGR1) polyclonal antibody (#CL488‐11739) from Proteintech. Images were obtained by an LSM880 fluorescence microscope from ZEISS.

### 4.6 Statistical analysis

The mean ± standard error of the mean was applied to express the results. When the data passed normality and equal variance tests, one‐way analysis of variance was performed to evaluate the significant differences (multiple comparisons by Bonferroni post hoc test and two groups comparisons by unpaired two‐tailed Student's *t*‐test). When the data could not pass the equal variance test, Welch correction was performed and the significant differences were evaluated by the Dunndett T3 post hoc test. Kaplan–Meier survival curves were applied to analyze the survival differences. The Statistical Package for the Social Sciences software (v22.0) was used to analyze all statistics. When *p* < 0.05, the statistical significance was defined.

## AUTHOR CONTRIBUTIONS

Y.J. conceived the project. T.T. and S.L. performed most of the experiments. H.L. developed the methodology and performed data analysis. Y.L., H.C., Y.Y., and G.C. performed gene KO mouse identification and animal experiments. B.X., Z.Y., Z.W., and L.L. performed cell culture and report gene assay. Y.J. designed and supervised the experiments and wrote the manuscript. All authors have read and approved the final manuscript.

## CONFLICT OF INTEREST STATEMENT

The authors declare that this study was conducted in the absence of any commercial or financial relationships that could be construed as a potential conflict of interest.

## ETHICS STATEMENT

All animal experiments conducted in this study received approval from the Animal Care and Use Committee at Southern Medical University, Guangzhou, China (approval number: L2018235).

## Supporting information

Supporting Information

## Data Availability

Upon request, the corresponding author will provide the relevant data.
