# Supplementary material for: LILAR, a novel long noncoding RNA regulating autophagy in the liver tissues of endotoxemic mice through a competing endogenous RNA mechanism
Source: MedComm (2020). 2023 Oct 10;4(5):e398. doi: 10.1002/mco2.398 (PMC10565381; doi:10.1002/mco2.398)
Supplement: Supplementary file 1 — Supporting Information [file MCO2-4-e398-s001.pdf]

Supplementary Data

***LILAR*, a novel long noncoding RNA regulating autophagy in the liver tissues of endotoxemic mice through a competing endogenous RNA mechanism**

Tian Tian[1, #], Shan Li[1, #], Haihua Luo[1, #], Yijing Li[1], Hanghang Chen[1], Ying Yang[1], Guangqin Chen[1], Bingyao Xie[1], Zhengzheng Yan[1], Zhenqi Wang[1], Lei Li[1], Yong Jiang[1,*]

[1]Guangdong Provincial Key Laboratory of Proteomics, State Key Laboratory of Organ Failure Research, Department of Pathophysiology, School of Basic Medical Sciences, Southern Medical University, Guangzhou 510515, China

#: These authors equally contributed to this work.

Running Head

LncRNA *LILAR* regulating autophagy through Atg13

***Correspondence to**:

Prof. Yong Jiang, M.D., Ph.D., Department of Pathophysiology, Southern Medical University, No.1023 Shatai South Road, Guangzhou 510515, Guangdong Province, China. Tel: +86-20-61648231; Fax: +86-20-61648231; E-mail: jiang48231@163.com.

Supplementary materials and methods

Generation and culture of bone marrow-derived macrophages (BMDMs)

BMDMs were generated according to previous reports.[1] In short, bone marrow cells were flushed out from tibia and femur and cultured in DMEM containing 10% FBS supplemented with penicillin (100 U/mL) and streptomycin (100 μg/mL) and 10 ng/mL macrophage colony-stimulating factor (M-CSF) (ThermoFisher Scientific, #RP-8615) at 37°C, 5% CO_2 for 7 days to differentiate into mature macrophages.

Materials

Lipopolysaccharide (LPS, #L2630) was obtained from Sigma Aldrich (Darmstadt, Germany). The antibodies used for Western blotting: anti-LC3B (#NB100-2220) was from NOVUS, anti-Atg13 (#13273), anti-SQSTM1/p62 (#5114), anti-mouse (#7076) and anti-rabbit (#7074) IgG HRP-linked antibody were from Cell Signaling Technology, GAPDH (MC4) mouse monoclonal antibody (#RM2002) was from Beijing Ray Antibody Biotech.

RNA-seq

A total RNA isolation kit (Biomarker, #RK02009) was used to isolate RNAs from liver tissues following the illustrations of the manufacturer. Before further experiments, the integrity, purity, and concentration of RNA were monitored and assessed to meet the subsequent requirements. For rRNA removal, the Ribo-Zero rRNA removal kit from Epicenter (Madison, WI, USA) was used. The Next Ultra Directional RNA Library Prep Kit (New England Biolabs) was utilized to generate the sequencing libraries following the illustrations of the manufacturer. After cluster generation by the TruSeq PE Cluster Kit (v3-cBot-HS), an Illumina HiSeq 4000 platform was used to sequence the library preparations.

Cell migration assay

We performed cell migration assays according to previous reports.[2] Corning Costar transwell plates (Corning, #3422, New York, USA) were used for cell migration assays according to the manufacturer's protocol. BMDMs were seeded in the upper chambers with FBS-free DMEM

media. The lower chamber was filled with DMEM containing 10% FBS as a chemo attractant. The cells were cultured at 37 °C in 5% CO_2 for 48 h. Then, the upper chambers were removed and the non-migrated BMDMs were wiped away. The 4% paraformaldehyde was used to fix migrated cells. After being stained with crystal violet (Beyotime, #C0121, Shanghai, China), the cells were observed under a microscope of EVO XL core AMEX1000 imaging system (ThermoFisher, Wilmington, DE, USA) and images were taken for cell counting with the cell counter plugin of the ImageJ software (Version 1.53u).

Phagocytosis assays

Phagocytosis assays were performed by pHrodo™ Red Zymosan Bioparticles (ThermoFisher, #P35364) according to the manufacturer's instructions. Briefly, Zymosan BioParticles conjugates of 0.5 mg/mL were resuspended for the medium replacement of the phagocytic cells. After incubation for 30 min, cells were acquired for analysis with the software (IDEAS, Version 6.0) on an ImageStream system (EMD Millipore, Washington, USA).

Protein extraction from liver tissues and cultured cells

RIPA buffer (ThermoFisher Scientific, #89900) with protease inhibitors (Roch, #11697498001) was utilized to extract proteins from liver tissues or HEK293T cells. Briefly, the tissue samples frozen in liquid nitrogen (approximately 5 mg) were crushed in a mortar and pestle. The samples were resuspended by RIPA buffer and sonicated with a VCX800 ultrasonic cell homogenizer (Sonics, CT, USA). HEK293T cells were collected by centrifugation at 500 × g for 5 min at 4°C, followed by resuspension with 300 μL of RIPA buffer and sonication as above. After centrifugation at 12,000 × g for 25 min at 4°C, the supernatant was collected for quantification of protein concentration by the bicinchoninic acid (BCA) method.

Cell culture

HEK293T cells were purchased from the American Type Culture Collection (ATCC, Maryland, USA). They were cultured in Dulbecco's modified Eagle's medium (DMEM, Gibco, #8120181), which was supplemented with 10% fetal bovine serum (FBS, Gibco, #16140071). HEK293T cells

were maintained at 37°C in humidified air containing 5% CO_2.

Histopathological examination

Liver tissues were isolated from the left lobe and the blood was removed by washing thrice in PBS. Following fixation in 4% paraformaldehyde (Biosharp, #BL539A) and subsequent paraffin embedding, slices of liver tissues were prepared for H&E staining. The injury score in each field was quantitated according to previous reports.[3]

Measurement of ALT, AST, and LDH

The serum levels of ALT, AST, and LDH were detected with specific kits for ALT (#C009-2), AST (#C010-2), and LDH (#A020-2) following the protocol of manufacturers (Jiancheng Bioengineering Institute, Nanjing, China).

Cytokine/chemokine quantitation

Quantification of serum cytokines and chemokines was performed using the LiquiChip system from Qiagen (Hilden, Germany) according to our previous reports.[4]

Overexpression of *LILAR* in mouse liver tissues

To achieve the overexpression of *LILAR* in mouse liver tissues, we commercially acquired an adeno-associated virus serotype 9 (AAV9) vector harboring *LILAR* (AAV9-*LILAR*) and an empty AAV9 vector as a negative control (AAV-NC) (Contract #GOSV0294502, Jikai Gene, Shanghai, China). The mice were treated with 1.5×10^{11} viral genomes (vg)/mouse recombinant AAV9 diluted in 200 μL normal saline via tail vein injection.

Isolation of total RNA and qRT-PCR

The manufacturer's protocol was followed for the isolation of total RNA from cells or tissues using TRIzol reagent (#15596026) supplied by Invitrogen (Carlsbad, CA, USA). The RNA concentration and quality were assessed using a NanoDrop spectrophotometer from ThermoFisher Scientific, USA. The reverse transcription of mRNA was performed by a ReverTra Ace™ qPCR RT Kit (#FSQ-101, TOYOBO, OSA, Japan) following the manufacturer's protocol. For microRNA, miR-705 was reversely transcribed using specific primers from RiboBio Co., Ltd. (Guangzhou,

China). The Applied Biosystems^{TM} 7500 real-time PCR system (CA, USA) was used to perform qRT-PCR analysis. The primers for mRNA are listed in Table S1. The Bulge-Loop miRNA qRT-PCR Primer Sets (RiboBio) were used for miR-705 (#MQPS0003068-1) and U6 (#MQPS0000002-1-100). The 18S rRNA and U6 were used as internal controls for the quantitation of mRNA and miRNA, respectively. The relative expression level of a gene was assessed utilizing the $2^{-\Delta\Delta CT}$ comparative method.

Flow cytometry analysis

Flow cytometry was performed as previously reported.[5] Splenic single cells were incubated with specific antibodies (Table S3) for staining at 4°C. After washing with PBS, cells were acquired for analysis with EDAS software (Version 6.0) on an ImageStream system (EMD Millipore, Washington, USA).

Transmission electron microscopy

Liver tissues were fixed with 2.5% glutaraldehyde at pH 7.4 for 1 h and processed for transmission electron microscopy (TEM) detection as previously described.[6] Briefly, after fixation and dehydration, an MT-6000-XL-RMC ultramicrotome from Boeckeler Instruments, Inc (Tucson, AZ) was used to obtain ultrathin sections (70–80 nm). Then, the sections were stained with uranyl acetate and lead citrate and examined with an H-7500 transmission electron microscope from Hitachi, Ltd (Tokyo, Japan).

Immunohistochemistry

Immunohistochemical analysis was performed according to previous reports.[7] Briefly, after deparaffinization and rehydration, the citrate buffer was used for antigen retrieval of the mouse liver tissue slices. Phosphate-buffered saline (PBS) containing 3% H_2O_2 was used to block endogenous peroxide. After blocking with 10% bovine serum albumin (BSA), the sections were incubated with monoclonal LC3B antibody (NOVUS, #NB100-2220) at 4°C overnight. After being treated with an avidin-biotin affinity system, the slices were stained with 3,3′-diaminobenzidine

(DAB) and hematoxylin for microscopic examination. The obtained pictures were analyzed by Image-Pro Plus (Version 6.0.0260).

Western blotting

Western blotting was performed as reported previously.[8] Samples containing equal amounts of proteins were separated by sodium dodecyl sulfate-polyacrylamide gel electrophoresis (SDS-PAGE) and transferred to polyvinylidene difluoride (PVDF) membranes from Millipore (#IPVH00010). The membranes were blocked with 5% BSA buffer (#V900933) from Sigma Aldrich (Darmstadt, Germany) and then incubated with the specific primary antibodies mentioned above. After 3 washes for 5 min with a Washing Buffer, i.e. Tris-buffered saline containing 0.1% Tween-20 (TBST), the membrane was incubated with horseradish peroxidase (HRP)-conjugated anti-mouse or rabbit secondary antibody. The protein bands were visualized by enhanced chemiluminescence (ECL) (ThermoFisher Scientific, #32109), and the images were captured using a ChemiDoc™ Imaging System provided by Bio-Rad (Hercules, CA, USA).

Dual-luciferase reporter assay

The pGL3-Atg13-3'UTR plasmid was constructed by inserting the Atg13-3'UTR sequence into the pGL3 vector (Promega, Madison, Wis, USA). The full sequence of *LILAR* was cloned into the pcDNA3 vector (pcDNA3-*LILAR*) to overexpress *LILAR* in 293T cells. Then, the miR-705 binding site-mutated pGL3-Atg13-3'UTR and pcDNA3-*LILAR* plasmids were constructed by PCR. The Renilla Luciferase constructed in the pRL-CMV vector was used as the reference reporter gene. The miR-705 mimic and negative control (mimic-NC) were obtained from RiboBio (Guangzhou, China). The reporter, WT, or mutated *LILAR* and miRNA mimics were transfected into 293T cells using Lipofectamine 3000 Reagent (Invitrogen, #L3000001) following the manufacturer's protocol. The transfection concentration was 300 ng for the pGL3 luciferase reporter vector, 10 ng for the pRL-CMV vector, and 50 nM for miRNA mimics. A Dual-Luciferase Reporter Assay System (Promega, Madison, Wis, USA) was utilized to perform luciferase assays. Ratios of Firefly luciferase and Renilla luciferase readings were evaluated in triplicate in each group.

RNA pulldown

RNA pulldown experiments were performed by using biotinylated *LILAR* (Bio-*LILAR*), Atg13-3'UTR (Bio-Atg13-3'UTR), and negative control (Bio-NC) based on previously described methods with some minor modifications. The biotinylated probes were prepared using a T7 RNA polymerase kit (Roche, #10881767001) following the manufacturer's instructions. RNA extracted from mouse livers was incubated overnight with a biotinylated probe of *LILAR*, Atg13-3'UTR, or negative control at 4°C. Subsequently, streptavidin-coated magnetic beads (ThermoFisher Scientific, #PI88817) were added and incubated for 2 h at 4°C. After 3 to 5 washes of the beads with incubation buffer, miR-705 in the pulldown mixture was measured by qRT-PCR.

Supplementary Figures

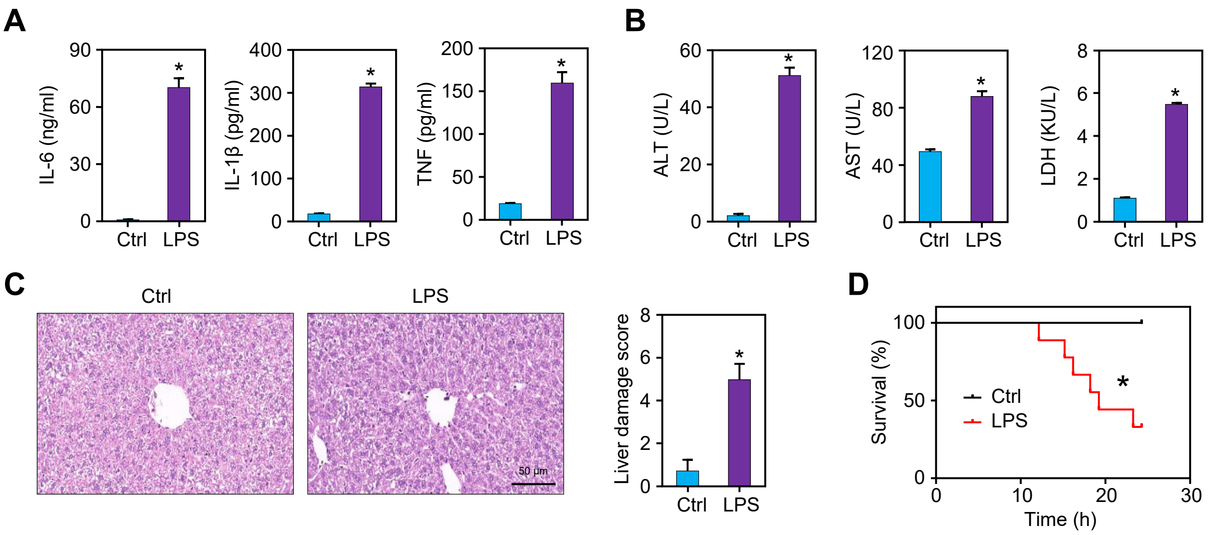

Figure S1. Assessment of liver injury model of mice induced by LPS. Mice were intraperitoneally injected with LPS (20 mg/kg) for 12 h or an equal volume of normal saline (NS) for control. **(A)** Protein quantitation of IL-6, IL-1β, and TNF in the serum of mice treated without or with LPS for 12 h. n = 3; *P < 0.05, compared with the control group. **(B)** The levels of ALT, AST, and LDH in the serum of mice treated without or with LPS for 12 h. n = 3; *P < 0.05, compared with the control group. **(C)** Histopathological evaluation of liver injury. H&E staining was performed as described in the methods and the amount of infiltration of inflammatory cells, hemorrhage, and hepatocyte necrosis were used as parameters to assess the liver injury. n = 4; *P < 0.05, compared with the control group. **(D)** The 24-hour survival rate of mice challenged with LPS. Statistical analyses were performed using the log-rank (Mantel-Cox) test. n = 10 for each group; *P <0.05, compared with control group.

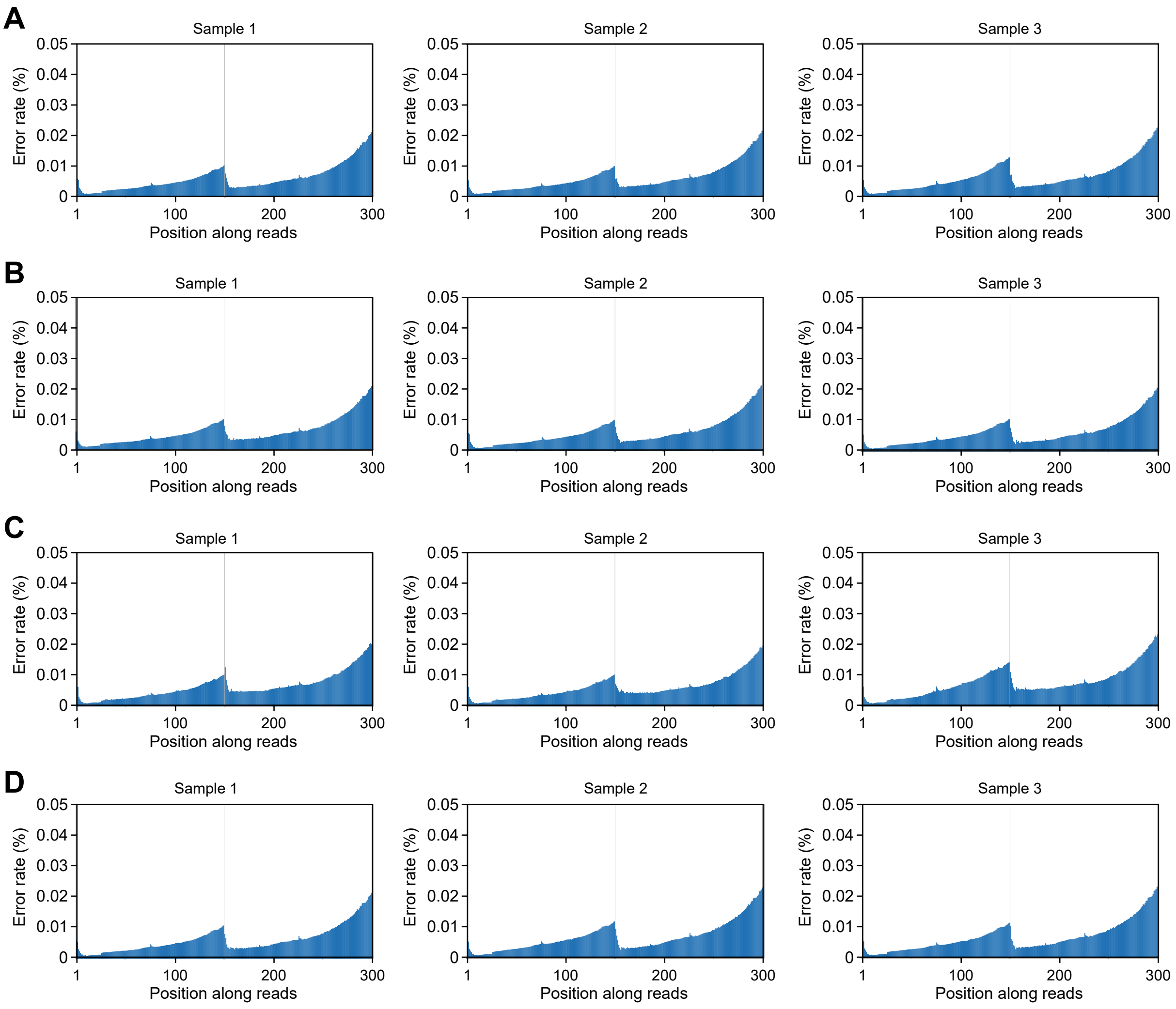

Figure S2. Quality assessment of RNA-sequencing data. (A-D) Error rate distribution along sequencing reads. There are a total of 4 groups, i.e. Ctrl (A), LPS 2h (B), LPS 8h (C), and LPS 24h (D) groups. Each group has three replicates (*n*=3).

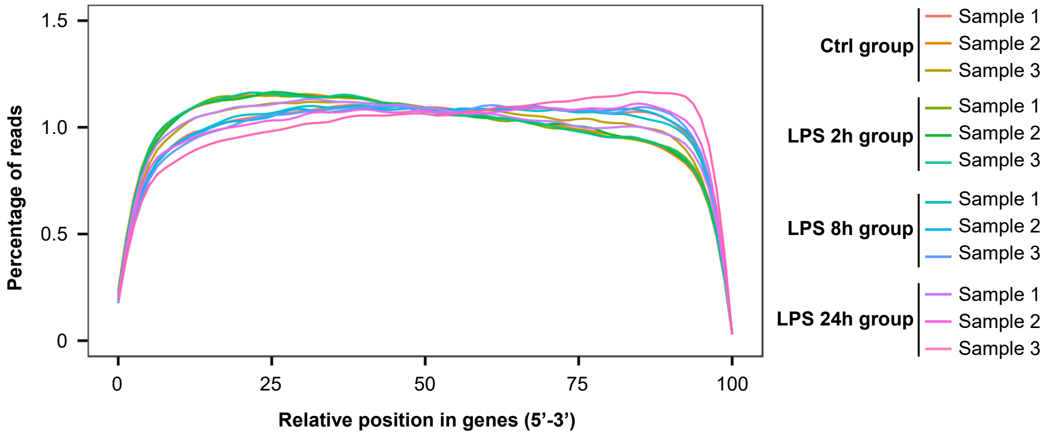

Figure S3. The distribution of mapped reads on mRNA sequences (5′-3′). Data reflects the percentage of mapped reads assigned to all regions of mRNA. The location of the normalized mRNA is on the horizontal (x) axis; the percentage of reads as compared to the total mapped reads for the position is on the vertical (y) axis. As the reference mRNA is different in length, each mRNA is divided into 100 intervals by length.

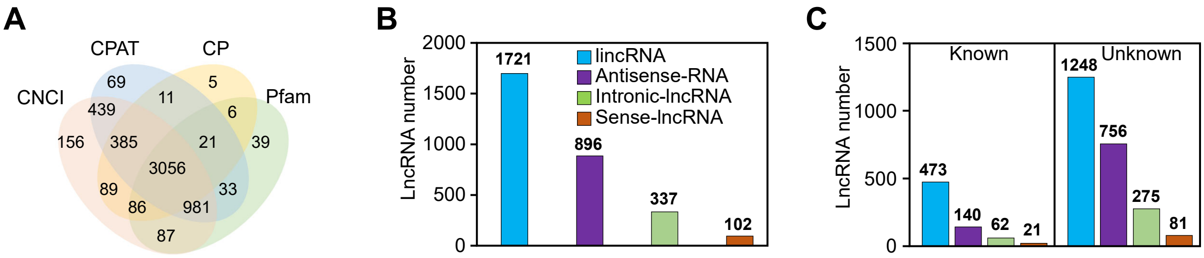

Figure S4. Comprehensive analysis of the characteristics of liver lncRNAs. (A) Venn diagram of lncRNAs predicted by four online software programs: Coding-Non-Coding Index (CNCI), Coding Potential Assessment Tool (CPAT), Coding Potential Calculator (CPC), and Pfam protein structure domain analysis. **(B)** Distribution of different kinds of lncRNAs, i.e., sense, antisense, intronic, and large intergenic noncoding RNAs in the liver tissues of mice treated without or with LPS for different times. **(C)** Distribution of known and unknown lncRNAs in the liver tissues of mice treated without or with LPS at different times.

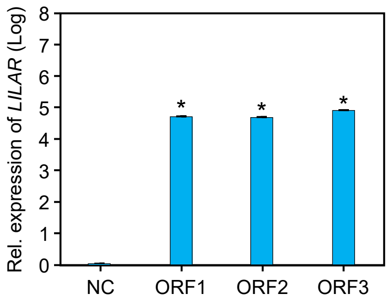

Figure S5. Detection of *LILAR* in 293T cells by qRT-PCR. The expression levels of lncRNA *LILAR* in 293T cells transfected with pcDNA3 plasmid carrying different ORFs of *LILAR*. Three different ORFs of *LILAR* were cloned into the eukaryotic expression vector pcDNA3 with a HA-tag. An empty pcDNA3 vector was used as negative control (NC). $n = 3$; * $P < 0.05$.

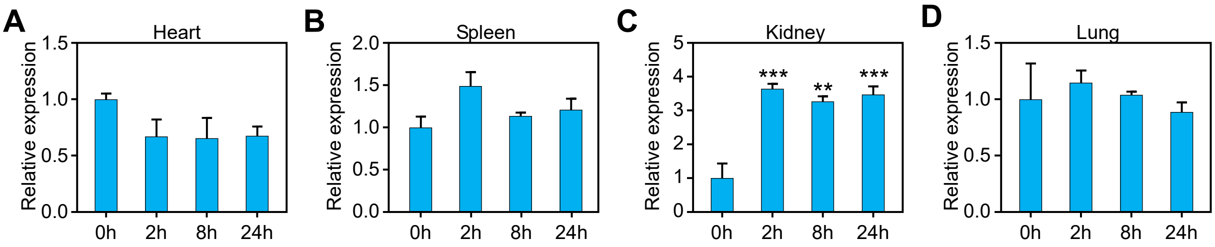

Figure S6. *LILAR* expression in different tissues of mice treated without or with LPS at different times. Heart (A), spleen (B), lung (C), and kidney (D) tissues were collected for the detection of *LILAR* transcripts by qPCR. $n = 3$; $^{**}P < 0.01$, $^{***}P < 0.001$.

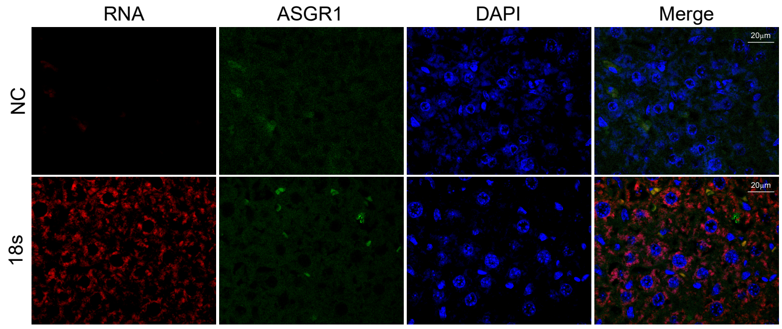

Figure S7. Fluorescence in situ hybridization analysis of control RNAs expressing in liver tissues. The expression of negative control (NC) and 18S RNAs in liver tissues of mice were detected by fluorescence in situ hybridization. Asialoglycoprotein receptor 1 (ASGR1) as an indicator of hepatocytes. Nuclei were labeled by DAPI.

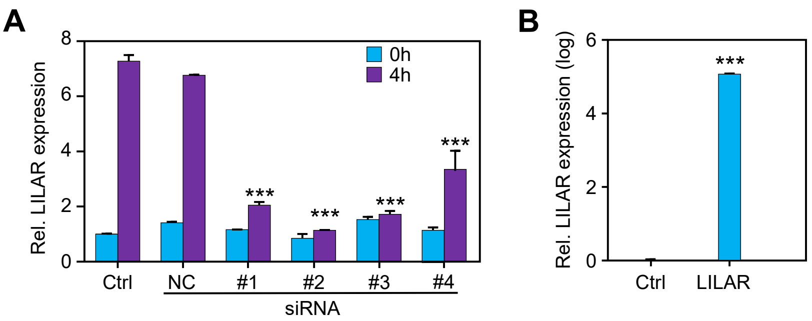

Figure S8. Validation of *LILAR* knockdown and overexpression in primary hepatocytes from mice. (A) The primary hepatocytes were transfected with negative control (NC) RNA or different siRNAs (#1, #2, #3, and #4) targeting *LILAR*. Twenty-four hours later, qRT-PCR was performed to quantitate the expression of *LILAR* in the primary hepatocytes treated without or with LPS for 4 h. n = 3; ***P < 0.001 compared with the control 4h group. The siRNA sequences can be found in Table S2. **(B)** The primary hepatocytes were transfected with pcDNA3 (control) or pcDNA3-*LILAR* plasmid. After 24 h, qRT-PCR was performed to quantitate the expression of *LILAR*. n = 3; ***P < 0.001 compared with control group.

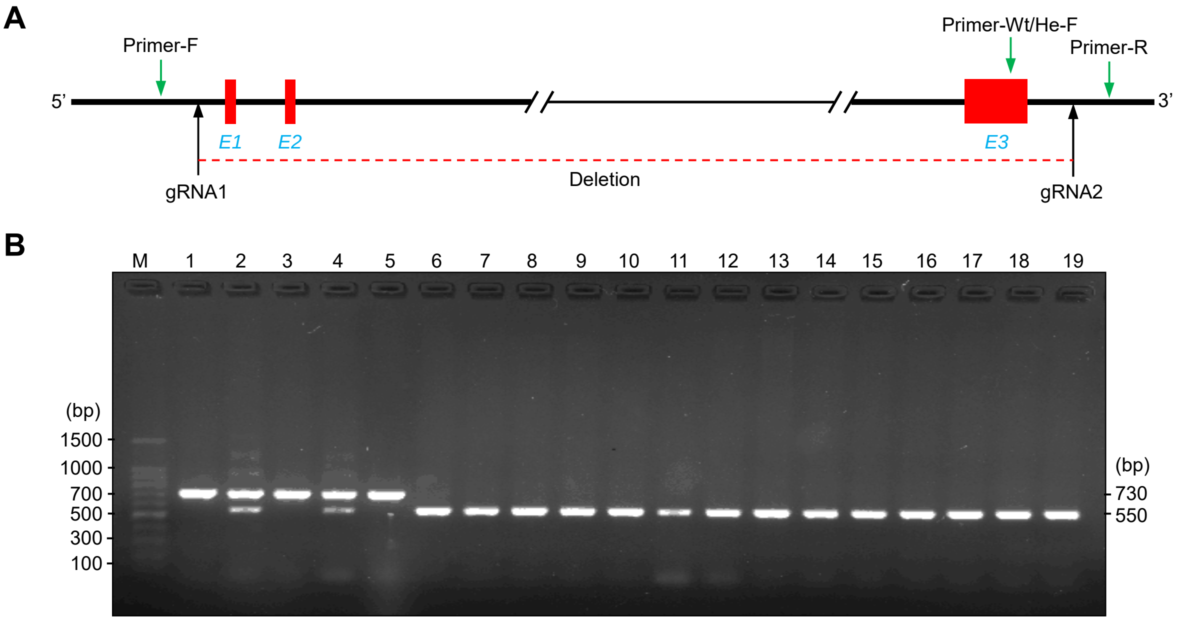

Figure S9. Construction of *LILAR* knockout (*LILAR*$^{-/-}$) mice with the CRISPR/Cas9 system. (A) Schematic gene targeting strategy to delete the three exons of lncRNA *LILAR* using the CRISPR/Cas9 system. Red boxes indicate annotated exons of *LILAR*. The dotted line shows the genomic region deleted by CRISPR)/Cas9-mediated genome editing. The location of the primers used to identify the successful knockout mice is indicated in the genome. **(B)** Agarose gel electrophoresis of PCR products of genomic DNA from three different genotype mice. Mice of wild type, *LILAR* deletion, or heterozygote were amplified with a forward primer (primer-F: CGGCAGTGAGGCTTCATTTCCTTA or primer-Wt/He-F: CCCGACATGGGCTTTCCTCTAG) and a reverse primer (primer-R: CAATGCCTACCTCTATTGGAAGGGAAG), resulting in a PCR band of 730 bp or 550 bp length, or double PCR bands with length of 730 bp and 550 bp, respectively.

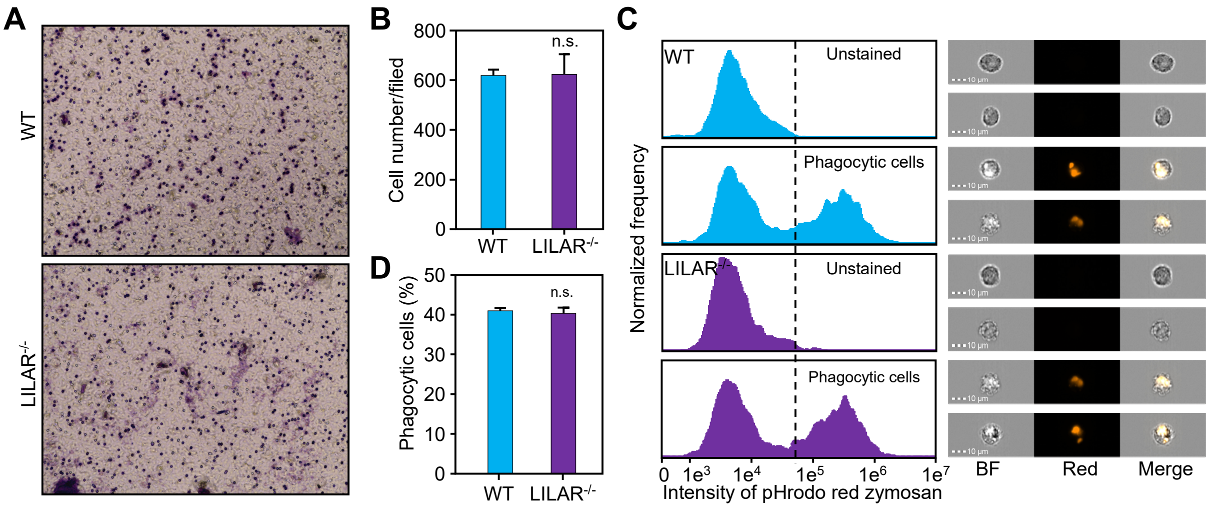

Figure S10. The deficiency of *LILAR* in mice does not affect the migration and phagocytosis of BMDMs.

(A and **B)** Migration assays of BMDMs were performed with Transwell plates. Representative images of migrated WT (upper) or *LILAR*$^{-/-}$(lower) BMDMs (A). Cell count of migrated macrophages (B). *n* = 3; n.s. = no significance. **(C** and **D)** Quantitation of BMDM phagocytosis by flow cytometry. Cultured BMDMs were stimulated with pHrodo red zymosan for 30 min, followed by phagocytosis analysis on the ImageStreamX Mk II Imaging Flow Cytometer. Fluorescence intensity histogram of the pHrodo red zymosan and representative images of phagocytic macrophages (C). The comparison was performed on the phagocytic percentages of macrophages from WT (upper) or *LILAR*$^{-/-}$(lower) mice (D). *n* = 3; n.s. = no significance.

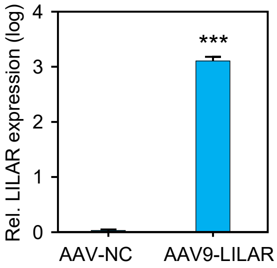

Figure S11. Validation of *LILAR* overexpression by AAV9-*LILAR* in the liver tissues from *LILAR* deficient mice. The *LILAR*$^{-/-}$ mice were subjected to tail vein injection of AAV9-*LILAR* or the negative control AAV9 vector (AAV-NC). Two weeks later, the liver tissues were collected from the *LILAR*$^{-/-}$ mice for the quantitation of *LILAR* expression by qRT-PCR. $n = 3$; ***$P < 0.001$ compared with NC group.

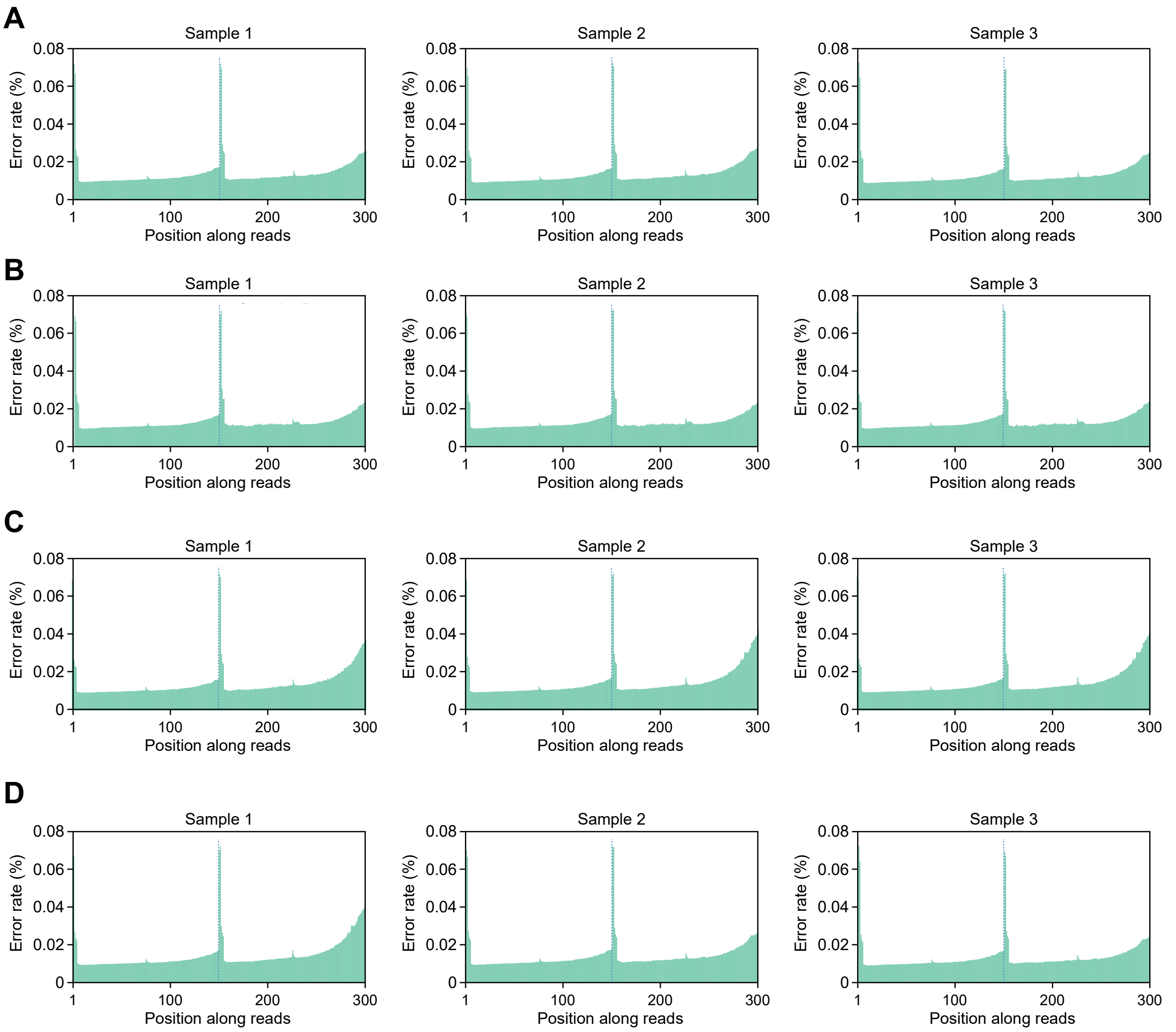

Figure S12. Quality assessment for sequencing data by error rate distribution along mRNA sequencing reads. Wild type (WT) and *LILAR*$^{-/-}$ mice were treated with LPS (20 mg/kg) or normal saline (NS) as the control for 4 h, liver mRNAs were obtained for RNA-sequencing. Each group has three replicates (*n* = 3). **(A)** WT mice treated with NS; **(B)** WT mice treated with LPS; **(C)** *LILAR*$^{-/-}$ mice treated with NS; **(D)** *LILAR*$^{-/-}$ mice treated with LPS.

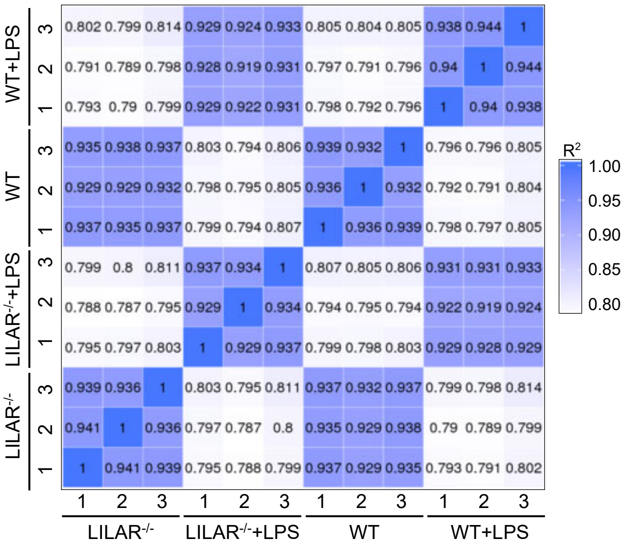

Figure S13. Pearson correlation coefficients between samples from WT and *LILAR* knockout (*LILAR*$^{-/-}$) mice treated with or without LPS. After treatment with or without LPS for 4h, mice were subjected to liver collection for RNA-seq. Coefficients between samples were acquired by Pearson correlation analysis.

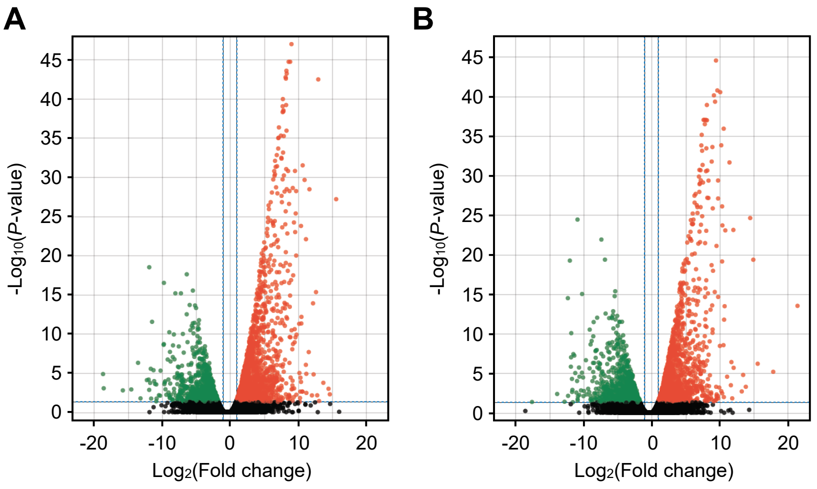

Figure S14. Volcano plots for differentially expressed genes in the livers of mice treated with LPS. (A) WT mice treated without or with LPS for 4 h. **(B)** *LILAR*$^{-/-}$ mice treated without or with LPS for 4 h. Red and green dots represent up- or down-regulated mRNAs by LPS, respectively.

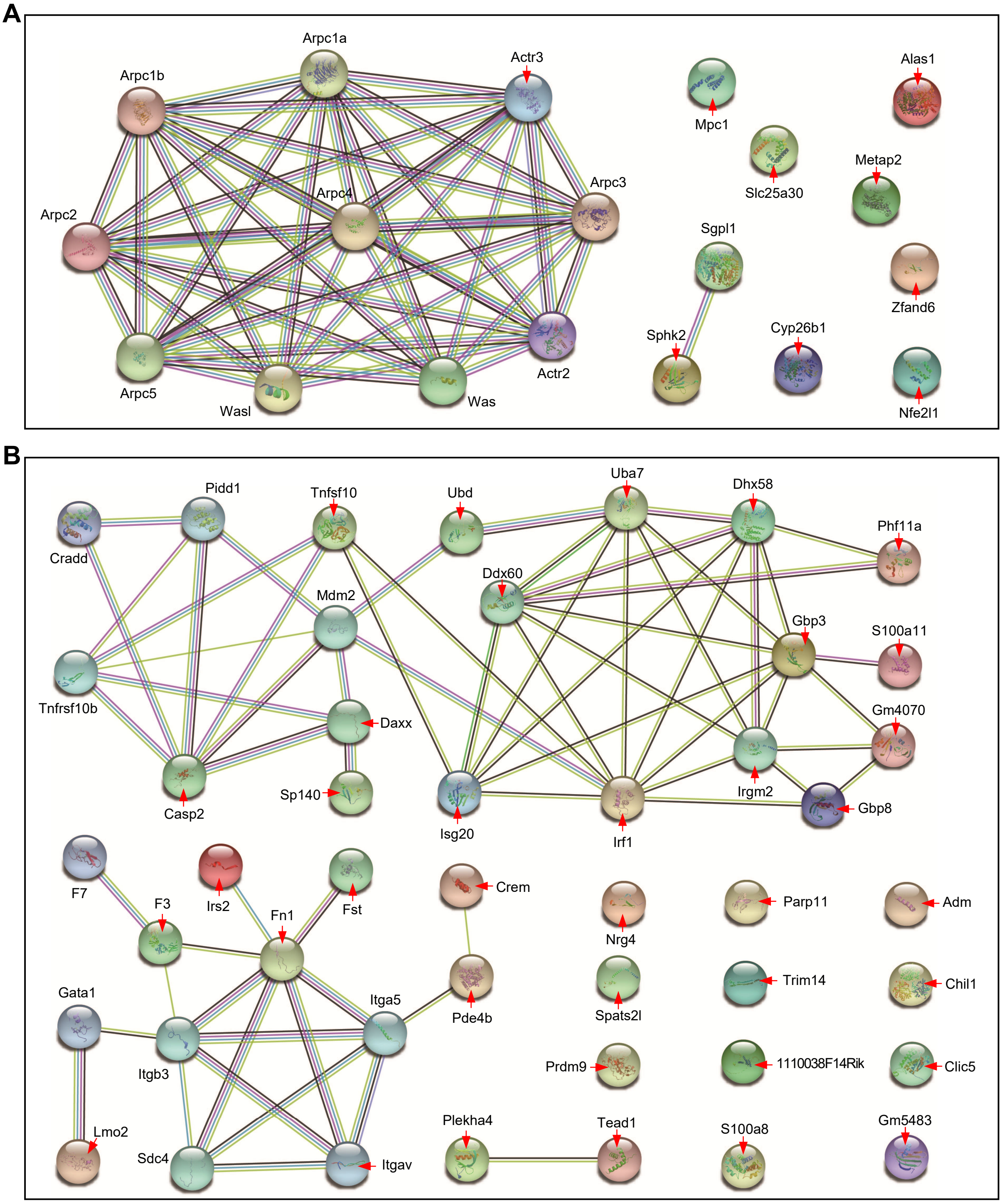

Figure S15. Protein-protein interaction analysis of products of Class I and Class II genes by STRING. (A and B) Products of Class I (A) and Class II genes (B) were analyzed by STRING with 1st shell filled with no more than 10 interactors. The genes from the Classes I and II were indicated by red arrows.

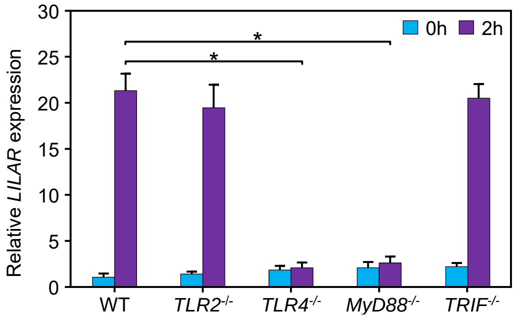

Figure S16. Relative expression levels of lncRNA *LILAR* in the liver tissues of TLR2 (*TLR2*$^{-/-}$), TLR4 (*TLR4*$^{-/-}$), MyD88 (*MyD88*$^{-/-}$), and TRIF (*TRIF*$^{-/-}$) gene deficiency mice. WT and gene knockout mice were stimulated with LPS (20 mg/kg) for 2 h and the expression of *LILAR* in the liver tissues was quantitated by qPCR. $n = 3$; * $P < 0.05$.

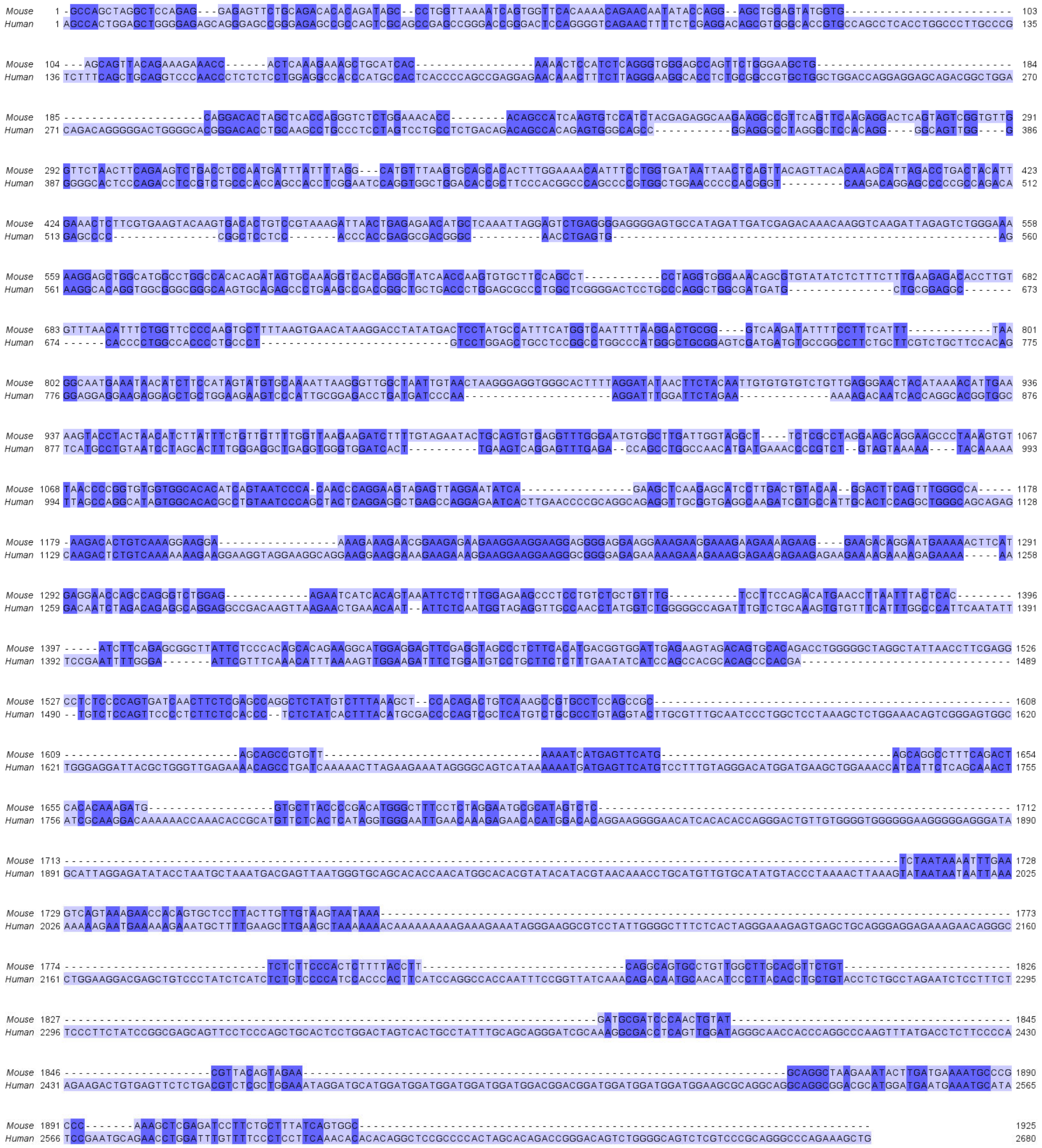

Figure S17. Sequence alignment between mouse *LILAR* and human ENSG00000283662. The two sequences were aligned using the default parameters of the MAFFT program (version 7.310) within Jalview software (version 2.11.2.7). In the alignment, regions that display conservation in both sequences are visually emphasized with a deep blue color highlighting.

Table S1. Primer sequences for qPCR.

Gene name	Primer	Sequence (5’-3’)
LILAR	Forward	TCACAAAACTCCATCTCAGG
	Reverse	TAACTGAGTTAATTATCACCAGGA
Atg13	Forward	AGAGACTGGTGATGCACATGCC
	Reverse	CCGTCCTTCACTGCTGTTAGAC
Il-6	Forward	TACCACTTCACAAGTCGGAGGC
	Reverse	CTGCAAGTGCATCATCGTTGTTC
Il1b	Forward	TGGACCTTCCAGGATGAGGACA
	Reverse	GTTCATCTCGGAGCCTGTAGTG
Tnf	Forward	GGTGCCTATGTCTCAGCCTCT
	Reverse	GCCATAGAACTGATGAGAGGGAG
18S	Forward	AGTCCCTGCCCTTTGTACACA
	Reverse	CGATCCGAGGGCCTCACTA

Table S2. Nucleotide sequences for FISH and siRNA

Application	Note	Sequence (5'-3')
**FISH probes**	NC	TGCTTTGCACGGTAACGCCTGTTTT
	18S	CTGCCTTCCTTGGATGTGGTAGCCGTTTC
	LILAR probe 1	AAACAGCAGACAGGAGGGCT
	LILAR probe 2	TGGGCGGGCATTTTCATCAA
	LILAR probe 3	AAAGCAGAAGGATCTCGAGC
**siRNAs target**	*LILAR* #1	CAGGCUCUAUGUCUUUAAATT
	LILAR #2	CGAGAUCCUUCUGCUUUAUTT
	LILAR #3	GUGGGAAACAGCGUGUAUATT
	LILAR #4	CACUGUCCGUAAAGAUUAATT
	NC	UUCUCCGAACGUGUCACGUTT

Table S3. Antibodies used for flow cytometry analysis.

Antigen	Fluorochrome	Catalog No.	Manufacturer
F4/80-Like Receptor	PE	563899	BD Bioscience
CD19	PerCP-Cy™5.5	551001	BD Bioscience
CD11c	FITC	557400	BD Bioscience
CD335 (NKp46)	PE	560757	BD Bioscience
CD11b	APC	553312	BD Bioscience
CD3	APC	565643	BD Bioscience
CD45R	APC	553092	BD Bioscience
CD4	FITC	553729	BD Bioscience
CD8	PE	553032	BD Bioscience

References

1. Toda G, Yamauchi T, Kadowaki T, Ueki K. Preparation and culture of bone marrow-derived macrophages from mice for functional analysis. *STAR Protoc*. 2021;2(1):100246.
2. Lan J, Luo H, Wu R, et al. Internalization of HMGB1 (High Mobility Group Box 1) promotes angiogenesis in endothelial cells. *Arterioscler Thromb Vasc Biol*. 2020;40(12):2922-2940.
3. Dawulieti J, Sun M, Zhao Y, et al. Treatment of severe sepsis with nanoparticulate cell-free DNA scavengers. *Sci Adv*. 2020;6(22):eaay7148.
4. Liu J, Wang J, Luo H, et al. Screening cytokine/chemokine profiles in serum and organs from an endotoxic shock mouse model by LiquiChip. *Sci China Life Sci*. 2017;60(11):1242-1250.
5. Lee Y, Wang Q, Shuryak I, Brenner DJ, Turner HC. Development of a high-throughput gamma-H2AX assay based on imaging flow cytometry. *Radiat Oncol*. 2019;14(1):150.
6. Du J, Zhang X, Han J, et al. Pro-inflammatory CXCR3 impairs mitochondrial function in experimental non-alcoholic steatohepatitis. *Theranostics*. 2017;7(17):4192-4203.
7. Langer R, Neppl C, Keller MD, Schmid RA, Tschan MP, Berezowska S. Expression Analysis of Autophagy Related Markers LC3B, p62 and HMGB1 Indicate an Autophagy-Independent Negative Prognostic Impact of High p62 Expression in Pulmonary Squamous Cell Carcinomas. *Cancers (Basel)*. 2018;10(9)
8. Yan Z, Luo H, Xie B, et al. Targeting adaptor protein SLP76 of RAGE as a therapeutic approach for lethal sepsis. *Nat Commun*. 2021;12(1):308.
